# How to Assess Shoulder Functionality: A Systematic Review of Existing Validated Outcome Measures

**DOI:** 10.3390/diagnostics11050845

**Published:** 2021-05-08

**Authors:** Rocio Aldon-Villegas, Carmen Ridao-Fernández, Dolores Torres-Enamorado, Gema Chamorro-Moriana

**Affiliations:** 1 Research Group “Area of Physiotherapy” CTS-305, Department of Physiotherapy, University of Seville, 41009 Seville, Spain; rocio.alvi@gmail.com (R.A.-V.); gchamorro@us.es (G.C.-M.); 2 Research Group “Women, Well-Being and Citizenship” SEJ066, Department of Nursing, University of Seville, 41930 Bormujos, Spain; dolores.torres@sjd.es

**Keywords:** shoulder, outcome measure, assessment scale, psychometrics properties, methodological quality, systematic review

## Abstract

The objective of this review was to compile validated functional shoulder assessment tools and analyse the methodological quality of their validations. Secondarily, we aimed to provide a comparison of the tools, including parameter descriptions, indications/applications, languages and operating instructions, to choose the most suitable for future clinical and research approaches. A systematic review (PRISMA) was conducted using: PubMed, WoS Scopus, CINHAL, Dialnet and reference lists until 2020. The main criteria for inclusion were that papers were original studies of validated tools or validation studies. Pre-established tables showed tools, validations, items/components, etc. The QUADAS-2 and COSMIN-RB were used to assess the methodological quality of validations. Ultimately, 85 studies were selected, 32 tools and 111 validations. Risk of bias scored lower than applicability, and patient selection got the best scores (QUADAS-2). Internal consistency had the highest quality and PROMs development the lowest (COSMIN-RB). Responsiveness was the most analysed metric property. Modified UCLA and SST obtained the highest quality in shoulder instability surgery, and SPADI in pain. The most approached topic was activities of daily living (81%). We compiled 32 validated functional shoulder assessment tools, and conducted an analysis of the methodological quality of 111 validations associated with them. Modified UCLA and SST showed the highest methodological quality in instability surgery and SPADI in pain.

## 1. Introduction

Shoulders are essential to human beings’ functionality. Their specific biomechanics make shoulders the most mobile joint complex in the body, providing upper limbs with mobility in the three axes of space [1].

Frequent shoulder dysfunctions are the third cause of musculoskeletal consultations in primary health care [2]. In fact, arthritis [3], rotator cuff (RC) injuries [4], shoulder instabilities [5] and fractures [6,7] constitute a large part of traumatology dysfunctions. Approximately 1% of the adult population in developed countries visit their doctor annually for shoulder pain [8]. For example, the incidence in the United Kingdom is 9.5 per 1000 inhabitants [9] and the annual prevalence in Spain is 70 to 200 cases per 1000 residents [10]. 

Currently, functional shoulder assessment methods are necessary to identify structural and/or biomechanical changes, and to link them to patients’ functional limitations and disabilities [11]. Furthermore, their use has increased in recent years [12], as they enable therapists and patients to work more objectively, standardise professional terms and develop and apply protocol treatments. All of this favours comparative analysis among the results obtained by the different interventions [13] and justifies the development of many functional shoulder assessment tools, with varying degrees of methodological quality and efficacy in the clinical setting.

However, the wide range of possibilities and the difficult access to outcome measures mean that the selection of the most appropriate way to assess shoulder function and disability could be a difficult task [11]. The content of each of them should be adapted to shoulder pathologies, symptoms, user characteristics and cultural, population and occupational contexts [11]. In addition, clinicians should also consider the quality methodological criteria, based on their validation studies and practical characteristics (e.g., duration and administration method), before making a decision [14].

Thus, because of the important role shoulders play in human beings’ functionality, the high incidence of shoulder dysfunctions, the importance of functional evaluation as well as the large number of tools and the difficulty in accessing them, the purpose of this systematic review was to compile the validated functional assessment tools and analyse the methodological quality of the validations associated with them. A second aim was to provide an operational comparison of the tools by means of parameter descriptions, indications, applications, languages and tool instructions for use, in order to choose the most suitable for future clinical and research approaches.

## 2. Materials and Methods

The method employed in this systematic review is based on the PRISMA statement [15]. The protocol was registered in the PROSPERO database (CRD42020218616). 

### 2.1. Data Sources and Search Strategy

An electronic search of PubMed, Web of Science (WoS), Scopus, Cumulative Index to Nursing and Allied Health Literature (CINAHL) and Dialnet (accessed on 11 January 2021) was conducted from inception through 2020, inclusive. The reference lists in this review and in each selected study were also considered to find other related articles. All papers that met the inclusion criteria were accepted.

Most the search terms used in this study came from Mesh (Medical Subject Headings). Other terms of interest were included due to their frequent use. The terms applied and the full list of search strategies are reported in Table 1.

### 2.2. Study Selection 

The included papers met the following inclusion criteria: original studies of validated functional shoulder assessment tools or validation studies (original or subsequently) associated with the identified scales, including physical tools or not; for human beings; validation studies in English, Spanish or French (outcome measures were included in any language). 

The reviewers, RA and DT, separately screened titles and abstracts of the search results to check if the studies met the pre-established inclusion criteria. GC solved the disagreements. The full texts of the studies that met the criteria were acquired and the causes for any exclusion at this stage were documented. 

### 2.3. Data Extraction

Data extraction was carried out by one reviewer (RA) and verified by a second reviewer (CR). Discrepancies between reviewers were resolved by a third reviewer (GC), who assessed the information independently. 

A pre-designed table details data regarding the shoulder assessment tools: authors, years, original validation studies and subsequent validation studies, indications/applications, countries of origin, languages, descriptions and instructions for use, observations (e.g., recommendations, location of the physical scale) and bibliographic references of interest. Another table shows the study population of the validations. In addition, a pre-designed comparative table includes data regarding the contents of items and components of each tool. The content percentages are represented in a complementary bar chart. 

The quality assessment was evaluated with two standardised tables. 

### 2.4. Assessment of Methodological Quality

Two assessment scales were used to evaluate the methodological quality of the validations included: the Quality Assessment of Diagnostic Accuracy Studies (QUADAS-2) [16] and the COnsensus-based Standards for the selection of health Measurement Instruments Risk of Bias checklist (COSMIN RB) [17].

QUADAS-2 is an evaluation scale for the diagnostic criteria of validation studies. It was employed to assess risk of bias and applicability. The seven items of this tool helped identify a “low”, “high” or “unclear” risk of bias in each domain, or concerns regarding applicability. QUADAS-2 is recommended by the Agency for Healthcare Research and Quality, the Cochrane Collaboration and the UK National Institute for Health and Clinical Excellence for use in systematic reviews regarding diagnostics [16].

COSMIN RB [17] was used as a tool to assess the adequacy and validity of the identified outcome measures [18]. This checklist consists of ten boxes, which correspond to ten metric properties, each of which contains items concerning aspects of design and statistical method. Each assessment was classified in a range of four levels: “very good”, “adequate”, “doubtful” and “inadequate” [17]. COSMIN RB does not take into account the metric properties that have not been applied in papers; that is, it does not evaluate the absence of metric properties negatively.

## 3. Results

### 3.1. Search Results

A total of 184,043 records were identified both on electronic databases and through manual search (reference lists). Following the removal of duplicates, 93,159 studies were screened by title, abstract and full text, rejecting them based on not being original studies of the validated functional shoulder assessment scales or not being validation studies (whether original or subsequent) associated with these outcome measures; not being for humans; and not being published in English, French or Spanish. After the screening, 85 studies were selected: 32 validated functional shoulder assessment scales and 73 validation studies including 111 validations associated with them. We would like to note that some validation studies validated more than one tool, and some original studies were not validation papers. 

**Figure 1:**
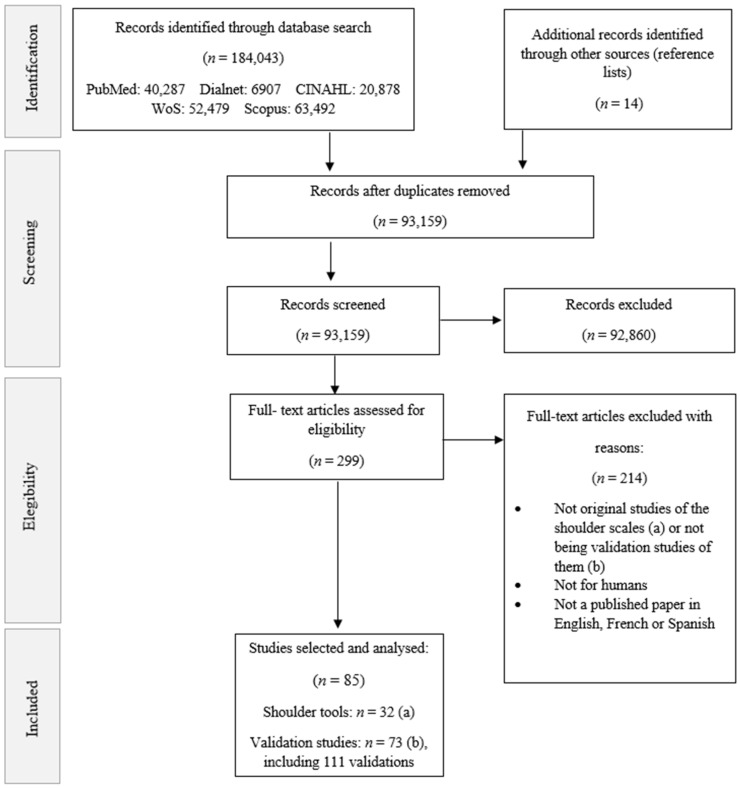
PRISMA flow diagram. Note: Some validations studies validated more than one assessment tool, and a few original studies were not validation papers.

Figure 1 presents the flow diagram of the study selection process based on the PRISMA protocol [15]. 

### 3.2. Characteristics of the Included Assessment Tools

Table 2 shows a summary of the descriptive data from the selected shoulder outcome measures: authors, years, original validation studies, other subsequent validation studies, indications or applications, countries of origin and languages, descriptions and instructions for use and observations (e.g., recommendations, location of physical scales).

### 3.3. Assessment of Methodological Quality

The results of the QUADAS-2 [16] and the COSMIN RB [273] for the 111 validations from 73 selected studies are shown in Table 3 and Table 4. 

The methodological quality results are summarised below. 

QUADAS-2. The *risk of bias* section obtained worse results than *applicability*.

- *Risk of bias*: *patient selection* stands out with 81/111 positive outcomes (72.97%). *Index test* and *reference standard* got 4/111 positive results (3.60%) and 70/111 unclear results (63.06%). The scope could not be evaluated in 37/111 cases (33.33%) in *index test*, *reference standard* and *flow and timing* due to the lack of a reference standard in the validation processes.
- *Concerns regarding applicability: patient selection* and *index test* got the best possible score in all validations (100%). The reference standard could not be evaluated in 37/111 cases (33.33%) as for the *risk of bias* section.

In relation to total QUADAS-2 scores, 4/111 validations (3.60%) stood out for obtaining 6 positive results in the QUADAS-2 and 22/111 validations (19.81%) achieved 5 results of “low” *risk of bias* or “low” concerns regarding *applicability*.

COSMIN RB.

- *Patient reported outcome measures (PROMs) development*: 22/111 validations (19.81%) were analysed and the score was “doubtful” in 6/22 cases (27.27%) and “inadequate” in 16/22 cases (72.73%).
- *Content validity* was addressed in 10/111 of the validations (9%), and the score was “adequate” in 1/10 cases (10%), “doubtful” in 4/10 cases (40%) and “inadequate” in 5/10 cases (50%).
- *Structural validity* was taken into account in only 6/111 validations, and 33.33% of the results were both “very good” and “inadequate”.
- *Internal consistency* was taken into account in 31/111 validations (27.92%), and 24/31 were very favourable, obtaining “very good” results (77.41%).
- *Reliability* was addressed in 58/111 validations (52.25%), and 41.38% of the results were both “adequate” and “inadequate”.
- *Measurement error* was calculated in 16/111 validations (14.41%), and many of its scores were “adequate” (76.47%).
- *Criterion validity* was considered for 16/111 validations (14.41%), and 12/16 stood out with “very good” results (75%).
- *Construct validity* was evaluated in 59/111 validations (53.15%), and 34/59 stood out for obtaining “very good” results (57.62%).
- *Responsiveness* was the most measured metric property. It was considered in 64/111 validations (57.65%). Of these, 14/64 obtained “very good” results (21.87%), 2/64 “adequate” (3.12%), 27/64 “doubtful” (42.18%), and 21/64 “inadequate” (32.81%).

Regarding the general COSMIN RB score, 9/111 validations obtained “very good” scores regarding the metric properties they addressed.

### 3.4. Indications/Applications, Transcultural Adaptations and Administration

In relation to the applications and indications, 13/32 tools (40.63%) [72,73,80,88,94,104,142,159,165,225,232,250,258] were initially designed to assess specific populations: shoulder pathologies like RC disease [73,142,159,258], instability [80,104,232], proximal humeral fracture [165] and osteoarthritis [250]; or surgical interventions like Bankart repair [88,94], RC disease repair [72] and total arthroplasty [225]. 

The populations in which the tools have been validated are listed below, differentiating among populations regarding symptoms/signs, pathologies and surgical treatments, whether general or specific techniques (Table 5).

Regarding the study populations, 58/111 validations (52.25%) were performed in a general population and 53/111 (47.75%) were carried out in a specific population (according to the pathology, surgical intervention or sign). A specific pathology was analysed in 28/111 cases (25.22%): RC disease in 12/111 validations (10.81%), shoulder instability in 7/111 (6.31%), humeral fracture in 6/111 (5.41%) and clavicle fracture, OA and adhesive capsulitis in 1/111 (0.90%). Surgical interventions were analysed in 24/111 validations (21.62%): surgical arthroplasty in 12/111 (10.81%), and RC repair and shoulder stabilisation procedures (as Bankart-type repairs or capsular shifts) in 6/111 (5.41%). A sign, scapula alata, was analysed in 1/111 studies (0.90%).

Regarding transcultural adaptations of rating scales, 17/32 outcome measures (53.12%) [12,19,44,58,82,88,94,104,111,132,142,167,203,206,232,250,258] were validated in other languages. The assessment tools that obtained the highest results in cross-cultural adaptation regarding languages were: Oxford Shoulder Score (OSS) [111] validated in 17 cases, Shoulder Pain and Disability Index (SPADI) [167] in 15, Western Ontario Shoulder Instability Index (WOSI) [232] in 12 and Western Ontario Rotator Cuff Index (WORC) [258] in 11.

In relation to the administration of the scale, 23/32 (71.87%) [12,58,63,72,80,100,104,111,129,132,142,159,165,167,196,203,206,216,223,230,232,250,258] are self-administered and 9/32 outcome measures (28.12%) [19,44,70,73,82,88,94,95,225] have to be administered by expert clinicians. 

### 3.5. Content Approached by Items and Components of the Tools

Table 6 shows the items and components of the outcome measures grouped by content. 

**Figure 2:**
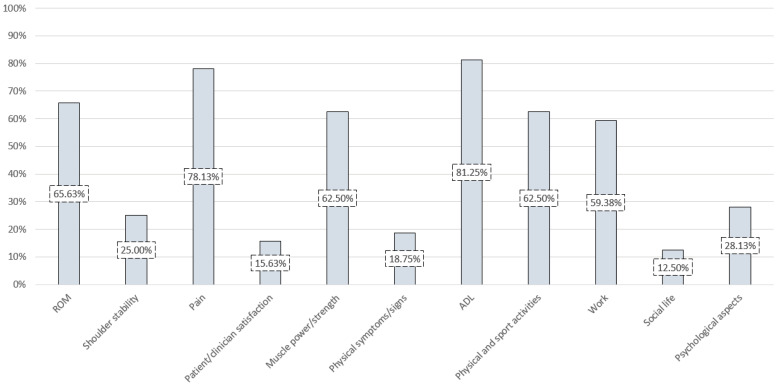
Frequency with which the scales consider specific topics. Abbreviations: ADL, activities of daily living; ROM, range of motion.

No topic was included in every tool, and no scale addressed all the contents presented. The frequency in which the subjects were considered by the evaluated tools is represented in percentages by means of a bar graph in Figure 2.

The contents addressed, in descending order of frequency, were: activities of daily living (ADL) (81.25%), pain (78.13%), range of motion (ROM) (65.63%), muscle power or strength (62.5%), physical and sport activities (62.5%), work (59.38%), psychological aspects (28.13%), shoulder stability (25%), physical symptoms or signs (18.75%) (compensations, weakness, stiffness, tenderness, atrophy, etc.), patient or clinician satisfaction (15.63%) and social life (12.5%).

## 4. Discussion

This systematic review compiled 32 validated functional assessment scales and analysed the methodological quality of 111 validations from 73 validation studies associated with said tools. Secondarily, an operational comparison of the methods was carried out to choose the most appropriate in each case, providing a detailed analysis of their characteristics: authors, years, validation studies, indications or applications, origins, languages, instructions for use and observations, as well as the topics addressed.

### 4.1. Methodological Quality

The QUADAS-2 [16] and the COSMIN RB [273] were used to assess methodological quality in a complementary way, which helped to determine the degree of reliability of the results obtained in the validations [17]. Regarding the QUADAS-2 [16], the patient selection domain obtained the best results because a large number of validations, such as that of Van der Windt [59], described the methodology used in this process and the patients included. However, two validations [144,160] included a convenience sample, increasing the risk of statistical bias in their results. The index test, reference standard and flow and timing domains could not be evaluated in 33% of the validations, as they did not include a reference standard. From a clinical perspective, the use of a reference standard is crucial, since it enables the comparison of the outcome measure that is being validated with a method of proven quality that can create scientific evidence. From a methodological perspective, the ideal validation study should include a blind and independent comparison between the tool to be validated and the reference standard, and both should be assessed in the same patient at the same time [16]. This was done by authors such as MacDermid et al. [170].

This review used the updated version of the COSMIN (2012) [274] (i.e., COSMIN RB (2018) [17]), developed exclusively for use in systematic reviews on outcome measures [17]. Consequently, it has led to a better assessment of the reliability of the results obtained, increased transparency, and therefore, a higher methodological quality of this study [17]. Additionally, the update has made it more intuitive and easier for reviewers to administer. However, including the new PROMs development section [17] resulted in the overall results being less favourable than those that would have been obtained with the COSMIN [274]. This is because the COSMIN RB makes it difficult for the validation studies that contained the tool design and development to obtain positive scores. Regarding the metric properties evaluated in this checklist, responsiveness was the most addressed. Despite this, this metric property obtained a large number of inadequate results because, among other reasons, the validations did not describe the intervention applied, as in Ge et al. [70], or the construct measured by the comparison instrument was not clear, as in the study by Razmjou et al. [47]. Regarding internal consistency, most of the validations obtained the highest possible score with the COSMIN RB. An example of this is the validation of Cook et al. [22], where the internal consistency statistic was calculated for each scale or subscale using Cronbach’s alpha. Regarding the design and development of the functional assessment methods, a large percentage of the validations obtained inadequate or doubtful results. This was because the researcher–patient interviews were not recorded or included notes, as in the validation of Razmjou et al. [159]. Furthermore, cases with small sample size—such as that of L’Insalata et al. [203], where the sample size was 30 patients—obtained an “inadequate” result. In order to obtain a “doubtful” result, 31 to 49 subjects are required and at least 50 are required for the result to be “adequate”. Optimising the sample size is essential for good methodological quality, since if the sample is too small the study not be able to detect an effect that is of interest, and if the sample is too large, it would suppose an unnecessary use of resources [275].

After comprehensive quality analysis using the QUADAS-2 [16] and the COSMIN RB [17], the results obtained using both tools contain inconsistencies. This occurred in the validation of Romeo et al. [89] of the Modified Rowe Shoulder Score [88], with a very favourable score using the QUADAS-2, but obtaining inappropriate results regarding reliability—the only metric property assessed by the COSMIN RB. The uncertainty about whether the patients’ health condition was the same at the time of each measurement determined the unfavourable reliability result. In contrast, the validations of Vascellari et al. [96] using the Modified University of California—Los Angeles Shoulder Scale (UCLA) [95] and the Simple Shoulder Test (SST) [206] obtained good results using both the QUADAS-2 and the COSMIN RB. This was interpreted as high reliability to assess arthroscopic Bankart repair or open Bristow-Latarjet procedure for recurrent anterior shoulder instability using the Modified UCLA [95] and the SST [206]. The same occurred with the validation of Van der Water et al. [165] using the Shoulder Function Index (SFInX) [165] and that of Bicer et al. [171] using the SPADI [167]. Thus, the SFInX [165] and the SPADI [167] are highly recommended in proximal humerus fractures and shoulder pain, respectively.

The Modified UCLA [95], the SPADI [167] and the SST [206] were the tools with the highest methodological quality according to the QUADAS-2 and the COSMIN RB. All of them obtained at least one validation with positive results: “low” risk of bias in 5/7 criteria (QUADAS-2), as well as “very good” and “adequate” (COSMIN RB). The aforementioned Modified UCLA [95], SPADI [167] and SST [206] were validated for a wide variety of dysfunctions, although they showed the highest quality for the assessment of surgical interventions for shoulder instability [96] using the Modified UCLA [95] and the SST [206] and shoulder pain [171] using the SPADI [167].

It should be noted that different factors need to be taken into consideration when choosing an assessment method. Therefore, a high level of methodological quality is neither the only characteristic to be taken into account, nor does it have to be the main one. Sometimes, the specificity of a scale regarding a population may be the key to the clinician’s decision making, as shown below. This is the case, for example, for scales designed specifically for a surgical intervention [72].

In relation to the four validations using the Modified UCLA [95], the most recent [96] (2018) obtained the highest quality due to scientific development over time. In the same way, the quality of the 10 validations using the SST [206] improved between 1996 [20] and 2020 [31]. These findings are linked to the current standards expected by prestigious scientific journals. In contrast, the 21 validations using the SPADI [167] did not evolve over time (1991 [167]–2020 [178]). In fact, the best quality was obtained in 2010 [171].

### 4.2. Indications/Applications and Cross-Cultural Adaptations 

Most of the functional assessment scales included have been applied for different shoulder injuries, as they are considered general assessment tools. However, as this study shows, some outcome measures were designed for a specific pathology, possibly due to their high incidence [276]. These include RC injuries [73,142,159,258], shoulder instabilities [80,104,232], proximal humerus fractures [165] and osteoarthritis [250]. Still, over the years, they have been validated and applied for different dysfunctions. This is the case of the WORC [258], which was originally designed only for RC injuries, but which was later validated for scapula alata [259] as well, expanding its application possibilities. On occasion, some specific scales have even been applied to other populations without having been validated—for example, the WOOS [250] was created for osteoarthritis and was subsequently used for proximal humerus fractures [253]. There are also tools for specific populations regarding symptoms/signs or general surgeries [26]. 

On the other hand, RC assessment methods stand out due to the large number of validations that support them [27,47,72,73,142,144,159,160,161,162,207,258]. In fact, these validations are both of their specific and general scales. However, it is noteworthy that another frequent dysfunction, adhesive capsulitis [277], does not have a specific validated tool, and only a general scale has been validated for this dysfunction (i.e., the SPADI) [167].

Many of the functional assessment scales included are known worldwide and have cross-cultural adaptations in other languages. The tool with the most cross-cultural adaptations is the OSS [111], as it is fast, practical, reliable, valid and clinically sensitive to changes. By contrast, despite having fewer adaptations to other languages, the CMS [44] is more often used [53] than the OSS [111], both at a clinical level and for scientific dissemination. 

Being aware of the wide variety of existing scales and their general or specific applications, linked to validations that ratify their effectiveness, makes it easier for the clinician to choose the most appropriate method in each case. 

### 4.3. Tool Administration

Regarding the administration of the assessment tools, the majority were self-administered by the patients (72%) following the authors’ instructions. Thus, the outcome measures used simple language users could understand, such as: “Is your shoulder comfortable with your arm at rest by your side?” [206] and “How much difficulty do you have sleeping because of your shoulder?” [232]. Furthermore, the Munich Shoulder Questionnaire [100] enables comprehension using images representing positions or actions. On the other hand, some scales require specialised shoulder clinicians to assess motion ranges [19,44,70,73,82,88,94,95,225], medical signs [19], muscle strength or power [19,44,70,82,95,225] and stability [19,88,94]. This can all be done through observation, palpation, instrumentation (goniometer), assessment tests (Daniels for strength, Apprehension Test for stability), etc. 

In recent decades, clinicians have tended to take patients’ perception into account [278], which improves communication between patient and clinician [279]. Indeed, the original version of the renowned UCLA [225] was modified to include the degree of patient satisfaction [95]. Complementing the objective data with this perception favours the evaluations and, therefore, decision making throughout the functional recovery process. This would justify the notable increase in the design and development of PROMs. 

### 4.4. Content Addressed by the Items and Components of the Tools

The items included in the scales are shown below in descending order of frequency. 

The ADL were the most considered component. Only 6 out of 32 outcome measures [12,88,94,129,196,223] did not address them. Including them is essential since they measure medical condition in terms of functionality [279]. In addition, specifically, “reaching above head level” was included by the vast majority of scales (81.25%).

The second most frequently included aspect was shoulder pain [19,44,58,70,72,73,80,82,88,95,100,104,111,129,132,142,167,196,203,223,225,230,232,250,258], possibly due to its high incidence in the population [280]. In particular, the Shoulder Pain Score [196] focuses solely on this topic. Night-time pain is highlighted specifically since the quality of sleep generally decreases in patients who suffer from it [281]. Lack of rest leads to the alteration of the abilities to perform the ADL, even having an impact on the emotional area. This justifies its consideration in the assessment tools. Indeed, Constant et al. [82] modified their original version to include night-time pain [82] among other items.

The ROM [12,19,44,58,63,70,73,80,82,88,94,95,100,129,132,142,203,206,223,225,232] was the third most considered topic. Regarding this, the great amplitude of the shoulder stands out [1]. This enables the performance of the necessary supracranial motions in the usual range of ADL. 

Physical and sports activities [12,44,80,82,88,94,100,104,129,132,142,165,203,206,216,223,230,232,250,258] and muscle strength and power [12,19,44,63,70,73,80,82,88,95,100,104,129,142,159,165,167,203,206,225,232] were the fourth most frequently considered topics. Both aspects are closely related. In fact, imbalance between external and internal rotation forces, as well as infraspinatus muscle atrophy, are common in volleyball players [282]. Furthermore, there is a clear link between certain sports and many shoulder injuries. For example, glenohumeral laxity and instability and scapular dyskinesia commonly affect swimmers [283]. RC disorders, especially subacromial impingement, are typical of golfers [284].

The direct relationship between shoulder impairment and appropriate work performance makes addressing the work area essential [12,44,80,82,88,94,100,104,111,132,142,159,203,206,216,223,232,250,258]. Shoulder disorders are the third most common cause of musculoskeletal consultations [2]. In particular, surgical interventions are directly linked with temporary work disabilities, and may even be permanent at times. This professional absenteeism not only causes socioeconomic losses but also affects the mental and emotional state [285]. 

Only a few tools addressed psychological aspects [58,100,104,129,142,230,232,250,258] and shoulder stability [19,80,88,94,104,129,223,232]. Shoulder stability and muscle strength are closely related—so much so that shoulder stability is improved through strength training [286]. Furthermore, stability together with a great shoulder ROM are essential for the adequate execution of ADL [287]. For their part, psychological factors are especially relevant and can lead to chronicity or modify the perception of the intensity of the pain and therefore the degree of dysfunction [288].

Physical signs and symptoms [19,129,159,232,250,258], degree of satisfaction [70,73,95,132,203] and social life [100,104,129,142] were the least addressed aspects, even though they also influence functionality. 

Despite the importance of the contents shown above, this review did not identify any functional assessment tool that included all of them. For this reason, a prospective study suggested by the authors would be the development of an outcome measure of methodological quality that includes this requirement.

### 4.5. Limitations and Strengths 

Regarding the limitations of this review, its extension—which resulted from the high number of identified tools and validations analysed—led the authors to exclude the methodological quality of cross-cultural adaptations. Even so, we decided to provide the references in order to make it easier for interested readers to find them.

As to its strengths, the paper compiled up to 32 validated shoulder outcome measures, providing a unique and useful document for the clinician to choose the most appropriate tool at all times. In addition, the methodological quality of the 111 validations associated with these scales was not only analysed using the COSMIN RB but supplemented with the QUADAS-2. This resulted in an even stronger basis for creating scientific evidence.

## 5. Conclusions

A necessary and practical compilation of 32 functional shoulder outcome measures was undertaken. The rating scales were systematically evaluated, and the methodological quality of 111 validations associated with these tools was analysed. An operational comparison of the outcome measures was also provided in order to facilitate the choice of the most appropriate for both clinical and research settings. The Modified University of California—Los Angeles Shoulder Scale and the Simple Shoulder Test showed the highest quality in the assessment of surgical interventions for shoulder instability, as did the Shoulder Pain and Disability Index for shoulder pain. The level of methodological quality is not the only factor to consider when selecting an assessment method. Specificity regarding the population, among other factors, could be decisive.

A large number of functional assessment tools were applied for different shoulder injuries, increasing the possibility of choice in their clinical application. The scales were mostly self-administered, clarifying the tendency to consider patients’ perceptions. Activities of daily living together with pain were the most addressed contents in the outcome measures.

## Figures and Tables

**Table 1:** Terms and search strategies.

Terms		Identifier
Scale OR scor* OR questionnaire OR test OR index OR assess* OR examination OR measure OR evaluation OR rating		1
Shoulder		2
**Database**	**Search strategy**	**Simplified strategy/** **Filters employed**
PubMed	*shoulder* AND (*scale* OR scor* OR *questionnaire* OR test OR *index* OR assess* OR *examination* OR *measure* OR *evaluation* OR rating)	1 AND 2 In humans
Web of Science	*shoulder* AND (*scale* OR scor* OR *questionnaire* OR test OR *index* OR assess* OR *examination* OR *measure* OR *evaluation* OR rating)	1 AND 2
Scopus	*shoulder* AND (*scale* OR scor* OR *questionnaire* OR test OR *index* OR assess* OR *examination* OR *measure* OR *evaluation* OR rating)	1 AND 2
CINAHL	*shoulder* AND (*scale* OR scor* OR *questionnaire* OR test OR *index* OR assess* OR *examination* OR *measure* OR *evaluation* OR rating)	1 AND 2
Dialnet	*shoulder* AND (*scale* OR scor* OR *questionnaire* OR test OR *index* OR assess* OR *examination* OR *measure* OR *evaluation* OR rating)	1 AND 2

Note: MESH terms are in italics. All databases were filtered by language. Papers written in English, Spanish and French were included. Abbreviations: scor*: score, scoring; assess*: assess, assessment.

**Table 2:** Characteristics of the included assessment tools.

Tools. Author, Years.	Original Validation Studies	Other Subsequent Validation Studies	Indications/ Applications	Country of Origin Languages	Description and Operating Instructions	Observations (Recommendation, Physical Scale, etc.)
1. AMERICAN SHOULDER AND ELBOW SURGEONS STANDARDIZED SHOULDER ASSESSMENT FORM (ASES) (Richards et al., 1994) [19]	Beaton et al., 1996 [20].	Beaton et al., 1998 [21], Cook et al., 2002 [22], Michener et al., 2002 [23], Oh et al., 2009 [24], Kemp et al., 2012 [25], Sciascia et al., 2017 [26], Dabija et al., 2019 [27], Vrotsou et al., 2019 [28], Gotlin et al., 2020 [29], Hou et al., 2020 [30], Baumgarten et al., 2020 [31].	Shoulder instability [25], total shoulder arthroplasty [26], rotator cuff (RC) tears [27], LHBT tenotomy [32], proximal humerus fracture [33], scapular dyskinesia and shoulder pain [34].	United States English [19], German [35], Italian [36], Arabic [37], Turkish [38], Dutch [39], Finnish [40], Portuguese [41], Spanish [42,43].	It consists of 2 sections: a patient self-evaluation and a clinician assessment. The patient self-evaluation form is divided into 2 parts: pain and instability, and activities of daily living (ADL). The clinician assessment portion consists of 3 components: range of motion (ROM), signs, and strength and instability. The shoulder score is derived by the following formula: (10 − Visual analogue scale pain score) x 5) + (5/3 x cumulative ADL score). The maximum score (100 points) indicates optimal state of the shoulder.	Beaton et al. [21] made a modification to ADL; 2 items were eliminated and 5 were added. The physical scale can be found in [19].
2. CONSTANT-MURLEY SCORE (CMS) (Constant et al., 1987) [44]	Conboy et al., 1996 [45].	Cook et al., 2002 [22], Angst et al., 2008 [46], Razmjou et al., 2008 [47], Rocourt et al., 2008 [48], Oh et al., 2009 [24], Kemp et al., 2012 [25], Ban et al., 2016 [49], Mahabier et al., 2016 [50], Sciascia et al., 2017 [26], James-Belin et al., 2018 [51].	Shoulder arthroplasty [46], RC disease [47], shoulder instability [25], clavicle fractures [49], humeral shaft fractures [50], subacromial pain [52].	United States English [44], Chinese [53], French [54], Portuguese-Brazilian [55], Italian [56], Arabic [57].	It consists of 13 items divided into 4 components: pain (15 points), ADL (20 points), ROM (40 points), and strength (25 points). The maximum score (100 points) indicates optimal state of the shoulder. Self-administered section and clinician assessment section.	CMS is one of the most commonly used international shoulder scoring scales [53]. The physical scale can be found in [44].
3. DUTCH SHOULDER DISABILITY QUESTIONNAIRE (DUTCH-SDQ) (Van der Heijden et al., 1996) [58]	Van der Windt et al., 1998 [59].	Van der Heijden et al., 2000 [60], Paul et al., 2004 [61].	Shoulder disorders [59,60], shoulder pain [61,62].	Netherlands English [59], Spanish [62].	It is composed of 16 questions in relation with shoulder functionality. Items scored by ticking a “yes”, “no”, or “not applicable” box if item does or does not describe patient. Items ticked “yes” are summed and normalized to 100. The maximum score (100 points) indicates the highest degree of disability. Self-administered.	The physical scale can be found in [59].
4. FLEXILEVEL SCALE OF SHOULDER FUNCTION (FLEX-SF) (Cook et al., 2003) [63]	Cook et al., 2003 [63].		Shoulder disorders [64], frozen shoulder syndrome [65], shoulder tightness [66], adhesive capsulitis [67], subacromial impingement syndrome [68], RC tears [69].	United States English [63].	It includes 3 tests that target low, medium, and high shoulder function. Each level is composed of 15 items. Each item is valued from 0 to 5. The patient performs 1 of these 3 levels of difficulty based on their lesion. The maximum score (60 points) indicates optimal state of the shoulder. Self-administered.	The physical scale can be found in [63].
5. FUDAN UNIVERSITY SHOULDER SCORE (Ge et al., 2013) [70]	Ge et al., 2013 [70].		Shoulder disorders [70], arthroscopic repair of the supraspinatus [71].	China English [70].	It is composed of 4 domains: pain (20 points), ADL (27 points), ROM and strength (32 points), and satisfaction of the patient and clinician (21 points). The maximum score (100 points) indicates the optimal state of the shoulder. Self-administered section and clinician assessment section.	Domains 1 and 2 comprise self-report assessments by patient, domain 3 is set up for clinician assessment. Section 4 is designed for both patient and clinician assessments. The physical scale can be found in [70].
6. FUNCTIONAL SHOULDER SCORE (FSS) (Iossifidis et al., 2015) [72]	Iossifidis et al., 2015 [72].		RC disorders [72].	United Kingdom English [72].	It is composed of 11 items divided into 2 categories: pain (1 item; 50 points) and ADL (10 items; 50 points). Each item has a possible score from 0 to 10 points. The maximum score (100 points) indicates the optimal state of the shoulder. e.g.,: Total score = (pain score x 5) + (ADL Score/2). Self-administered.	The physical scale can be found in [72].
7. KOREAN SHOULDER SCORING SYSTEM (KSS) (Tae et al., 2009) [73]	Tae et al., 2009 [73].		RC disorders [73], RC repair [74,75,76], acromioclavicular joint dislocation [77], humeral fracture [78], adhesive capsulitis [79].	Korea English [73].	It is composed of 5 domains: function (ADL) (30 points); pain (20 points); satisfaction (10 points); ROM (20 points); and muscle power, consisting of strength (10 points) and endurance (10 points). The maximum score (100 points) indicates optimal state of the shoulder. Self-administered section and clinician assessment section.	The physical scale can be found in [73].
8. MELBOURNE INSTABILITY SHOULDER SCALE (MISS) (Watson et al., 2005) [80]	Watson et al., 2005 [80].		Shoulder instability [80,81].	United States English [80].	It is composed of 22 items divided into 4 subgroups: pain (15 points), instability (33 points), function (32 points), and occupation and sporting demands (20 points). The maximum score (100 points) indicates optimal state of the shoulder. Self-administered.	The scale contains a personal data sheet and medical information of interest. The physical scale can be found in [80].
9. MODIFIED CONSTANT-MURLEY SCORE (CMS) (Constant et al., 2008) [82]	Van der Water et al., 2014 [83].		Proximal humeral fracture [83], shoulder impingement syndrome [84], shoulder pain [85], RC tears [86].	United States English [82], Danish [84], Greek [85], Turkish [87].	It consists of 13 items divided into 4 components: pain (15 points), ADL (20 points), ROM (40 points), and strength (25 points). The maximum score (100 points) indicates optimal state of the shoulder. Self-administered section and clinician assessment section.	Is a modification of the Constant-Murley Score [44]. Constant modified how to measure pain (VAS added), ADL (questions included), ROM (more indications), and strength (guidelines for correct measures). The physical scale can be found in [82].
10. MODIFIED ROWE SHOULDER SCORE (MRS) (Rowe et al., 1981) [88]	Romeo et al., 1996 [89].		Shoulder subluxation [88], shoulder instability [89], anterior capsulolabral reconstruction [90], proximal humerus fractures [91], posterior shoulder dislocation [92], and SLAP lesion [93].	United States English [88], Portuguese [93].	It consists of 4 components: function (50 points), pain (10 points), stability (30 points), and ROM (10 points). The maximum score (100 points) indicates optimal state of the shoulder. Interpretation: excellent (90–100 points), good (70–89 points), fair (40–49 points), and poor (<39 points). Self-administered section and clinician assessment section.	Is a modification of the Rowe Scale [94]. The items and the interpretation of both scales are different. The physical scale can be found in [88].
11. MODIFIED UNIVERSITY OF CALIFORNIA—LOS ANGELES SHOULDER SCALE (UCLA) (Ellman et al., 1986) [95]	Cook et al., 2002 [22].	Oh et al., 2009 [24], Van de Water et al., 2014 [83], Vascellari et al., 2018 [96].	Shoulder disorders [22], shoulder surgery [24], proximal humeral fractures [83], anterior shoulder instability surgery [96], RC repair and proximal humeral fracture osteosynthesis [97], impingement syndrome [98].	United States English [95], Italian [99].	It is composed of 5 components: pain (10 points), function (10 points), ROM (5 points), muscular strength (5 points), and patient satisfaction (5 points). The maximum score (35 points) indicates optimal state of the shoulder. Interpretation: excellent evaluation (35–34 points), good (33–29 points), fair (27–21 points), and poor result (<20 points). Self-administered section and clinician assessment section.	The physical scale can be found in [95].
12. MUNICH SHOULDER QUESTIONNAIRE (MSQ) (Schmidutz et al., 2012) [100]	Schmidutz et al., 2012 [100].		Shoulder disorders [100], reconstruction of proximal humerus fractures [101], subacromial impingement [102], dislocated fracture of the lateral clavicle [103].	Germany English [100].	It consists of 30 items divided into 6 domains: ROM (5 items; 50 points), power of the shoulder (1 item; 24 points), pain (6 items; 60 points), work and ADL (9 items; 90 points), recreational activities/sports (6 items; 60 points), and social life (3 items; 30 points). The maximum score (314 points) indicates optimal state of the shoulder. The score can be reported as a percentage of normal by subtracting the total from 314, dividing by 314, and multiplying by 100. e.g.,: (314 − total score/314) x 100. Self-administered.	It consists of 3 parts: cover sheet, objective section, and subjective assessment. The physical scale can be found in [100].
13. OXFORD INSTABILITY SCORE (OIS) (Dawson et al., 1999) [104]	Dawson et al., 1999 [104].	Van der Linde et al., 2017 [105].	Shoulder instability [105,106], arthroscopic Bankart repair [107], SLAP lesion [108].	United Kingdom English [104], Dutch [106], Italian [109], Turkish [110].	It is composed of 12 questions in relation to shoulder instability (5 points), pain (10 points), occupational sphere (5 points), ADL (20 points), physical and sport activities (5 points), social life (5 points), and psychosocial aspects (10 points). The maximum score (60 points) indicates the highest degree of disability. Self-administered.	The physical scale can be found in [104].
14. OXFORD SHOULDER SCORE (OSS) (Dawson et al., 1996) [111]	Dawson et al., 1996 [111].	Van de Water et al., 2014 [83].	Impingement or tendinitis of the shoulder [112], disorders of the RC [113], proximal humerus fractures [113], shoulder pain [114], shoulder disorders, rheumatoid arthritis [115], frozen shoulder [116].	United Kingdom English [111], German [112], Romanian [113], French [114], Portuguese-Brazilian [115], Portuguese [117], Polish [118], Turkish [119], Korean [120], Chinese [121], Italian [122], Dutch [123], Persian [124], Danish [125], Norwegian [126], Arabic [127], Spanish [128].	It is composed of 12 items divided into 2 subscales: pain (20 points) and ADL (40 points). Each item is rated from 1 to 5 points. The maximum score (60 points) indicates the highest degree of disability. Self-administered.	The physical scale can be found in [111].
15. PEDIATRIC/ ADOLESCENT SHOULDER SURVEY (PASS) (Edmonds et al., 2017) [129]	Edmonds et al., 2017 [129].		Shoulder disorders [129], shoulder instability [130], glenoid labral pathology [131].	United States English [129].	Consists of 13 questions that assess symptoms, limitations, need for compensatory mechanisms, and emotional distress. Each question is provided on a 0 to 5 scale (questions 2, 4, 9, 10, 12, 13) or 0 to 10 scale (questions 1, 3, 5–8, 11). Once reverse scoring is applied to items 1 through 9, the reverse scores from items 1 through 9 are summed together with the actual scores from items 10 through 13. The formula for the total score is: SUM (reverse score 1–9, 10–13)/100. The maximum score (100 points) indicates optimal state of the shoulder. Self-administered.	The PASS was developed because most of the adult-age questionnaires ask questions that are not age appropriate. The physical scale can be found in [129].
16. PENN SHOULDER SCORE (PSS) (Leggin et al., 1999) [132]	Cook et al., 2001 [133].	Leggin et al., 2006 [134].	Shoulder disorders [135], subacromial pain syndrome [136], reverse shoulder arthroplasty [137], RC repair [138], scapular dyskinesis [139], shoulder pain [140].	United States English [134], Turkish [135], Portuguese [140], Brazilian [141].	It consists of 24 items divided into 3 components: pain (30 points), satisfaction (10 points), and function (60 points). The pain subscale consists of 3 pain items. All are based on a 10-point numeric rating scale. Patient satisfaction is also assessed with a 10-point numeric rating scale. The function subsection is based on a sum of 20 items, each with a 4-point Likert scale. The maximum score (100 points) indicates optimal state of the shoulder. Self-administered.	The PSS can be used in the aggregate or each subscale individually. The physical scale can be found in [134].
17. ROTATOR CUFF QUALITY OF LIFE (RC-QOL) (Hollinshead et al., 2000) [142]	Hollinshead et al., 2000 [142].	Razmjou et al., 2006 [143], Eubank et al., 2017 [144].	RC disease [142,144], impingement syndrome, RC repair, acromioplasty, or decompression surgeries [143], full-thickness RC tears [145], latissimus dorsi tendon transfer and partial cuff repair in irreparable postero-superior RC tear [146], chronic RC tear [147].	Canada English [142], Italian [148], Chinese [149,150], Turkish [151], German [152], Spanish [153].	It is composed of 34 items divided into 5 domains: symptoms and physical complaints (16 items), sport/recreation (4 items), work-related concerns (4 items), lifestyle issues (5 items), and social and emotional issues (5 items). Each item has a possible score from 0 to 100 (100 mm visual analogue scale). The maximum score (100 mm) indicates optimal state of the shoulder. Self-administered.	The instrument provides instructions to the patients. The physical scale can be found in [142].
18. ROWE SCALE (Rowe et al., 1978) [94]	Romeo et al., 1996 [89].	Oh et al., 2009 [24].	Shoulder instability [89], shoulder surgery [24], anterior shoulder luxation [154], anterior shoulder reconstruction [155], Latarjet surgery for traumatic anterior shoulder instability [156], arthroscopic Bankart repair for shoulder instability [157].	United States English [94], Portuguese [158].	It is composed of 3 components: shoulder stability (50 points), ROM (20 points), and function (30 points). The maximum score (100 points) indicates optimal state of the shoulder. Interpretation: excellent evaluation (90–100 points), good (75–89 points), fair (74–51 points), and poor evaluation (50–0 points). Self-administered section and clinician assessment section.	There are 4 different Rowe score versions. This is the original version of the Modified Rowe Scale. This is the first scale developed for this purpose. The physical scale can be found in [94].
19. SHORT WESTERN ONTARIO ROTATOR CUFF INDEX (SHORTWORC) (Razmjou et al., 2012) [159]	Razmjou et al., 2012 [159].	Dewan et al., 2016 [160], Dewan et al., 2018 [161], Furtado et al., 2020 [162].	RC repair [159,160,161], RC pathology [162].	Canada English [159].	It consists of 7 items, including all items from the WORC work and lifestyle domains except the one relating to roughhousing. Each item has a possible score from 0 to 100 (100 mm visual analogue scale) and these scores are added to give a total score from 0 to 700 points. The maximum score (700 points) indicates the highest degree of disability. The score can be reported as a percentage of normal by subtracting the total from 700, dividing by 700, and multiplying by 100. e.g.,: (700 − total score/700) x 100 Self-administered.	If answers to 10% of questions are missing for an index, the index is considered to be missing completely. The physical scale can be found in [159].
20. SHOULDER ACTIVITY RATING SCALE (SARS) (Brophy et al., 2005) [12]	Brophy et al., 2005 [12].		Shoulder disorders [12,163], total shoulder arthroplasty [164].	United States English [12], Persian [163].	It is a numeral sum of scores for physical activities: carrying objects 8 pounds or heavier by hand, handling objects overhead, weight training with arms, swinging motion, and lifting objects 25 pounds or heavier. Each of the 5 activity items was scored from never performed (0 points) to daily (4 points). Two additional multiple choice questions provide a score assessing participation in contact and overhead sports. The maximum score (20 points) indicates optimal state of the shoulder. Self-administered.	The physical scale can be found in [12].
21. SHOULDER FUNCTION INDEX (SFInX) (Van de Water et al., 2015) [165]	Van de Water et al., 2015 [165].	Van de Water et al., 2015 [166].	Proximal humeral fractures [165,166].	Australia English [165].	It is composed of 13 questions that evaluate shoulder function. The scoring categories for 5 items are “able” or “unable”, and 8 items also have a middle “partially able” category, which is chosen when compensation is used to complete the task. Total raw scores are converted to a 0–100 interval level SFInX score using the conversion table on the assessment form. The maximum score (100 points) indicates optimal state of the shoulder. Self-administered.	The physical scale can be found in [165].
22. SHOULDER PAIN AND DISABILITY INDEX (SPADI) (Roach et al., 1991) [167]	Roach et al., 1991 [167].	Beaton et al., 1996 [20], Heald et al., 1997 [168], Beaton et al., 1998 [21], Roddey et al., 2000 [169], Cook et al., 2001 [133], Cook et al., 2002 [22], Paul et al., 2004 [61], MacDermid et al., 2006 [170], Angst et al., 2008 [46], Bicer et al., 2010 [171], Staples et al., 2010 [172], Hill et al., 2011 [173], Riley et al., 2015 [174], Jerosch-Herold et al., 2017 [175], Thoomes de Graaf et al., 2017 [176], James-Berlin et al., 2018 [51], Vascellari et al., 2018 [96], Riley et al., 2019 [177], Dabija et al., 2019 [27], Boake et al., 2020 [178].	Shoulder disorders [168], shoulder pain [171], adhesive capsulitis [172], RC disease [51], shoulder arthroplasty [179].	United States English [167], German [179], Arabic [180], Chinese [181,182], Danish [183], Dutch [184], Greek [185,186], Italian [99,187], Korean [188], Nepali [189], Slovene [190], Thai [191], Indian [192], Japanese [193], Spanish [194].	It contains 13 items that assess two domains: a 5-item subscale that measures pain and an 8-item subscale that measures disability. Each subscale is summed and transformed to a score out of 100. A mean is taken of the two subscales. The maximum score (100 points) indicates the highest degree of disability. Self-administered.	There are 2 versions of the SPADI; the original version has each item scored on a visual analogue scale (VAS). The second version has items scored on a numerical rating scale (NRS) [195]. The physical scale can be found in [167].
23. SHOULDER PAIN SCORE (SPS) (Winters et al., 1996) [196]	Winters et al., 1996 [196].		Shoulder arthroplasty [197], periarthritis humeroscapularis [198], arthroscopic RC repair [199], subacromial impingement [200], laparoscopic gastric bypass [201], oral squamous cell carcinoma [202].	Netherlands English [196].	It contains 7 items about pain: pain at rest, pain in motion, nightly pain, sleeping problems caused by pain, incapability of lying on the painful side, degree of radiation, and numerical pain scale. Each item was scored from none (0 points) to severe/past the elbow (4 points). The maximum score (28 points) indicates the highest degree of disability. Self-administered.	The physical scale can be found in [196].
24. SHOULDER RATING QUESTIONNAIRE (SRQ) (L’Insalata et al., 1997) [203]	L’Insalata et al., 1997 [203].	Paul et al., 2004 [61].	Shoulder pain [61], shoulder disorders [204,205], shoulder pain or limitation of function [188].	United States English [203], Dutch [204], Portuguese [205], Korean [188].	It is composed of 21 items divided into 6 groups: global evaluation (domain score multiplied by 1.5; score range, 0 to 15 points), pain (domain score multiplied by 4; score range, 8 to 40 points), ADL (domain score multiplied by 2; score range, 4 to 20 points), recreational and athletic activities (domain score multiplied by 1.5; score range, 3 to 15 points), work (domain score multiplied by 1; score range, 2 to 10 points), and satisfaction (points not included in the total score). The global assessment domain consists of a 10 cm visual analogue scale. This scale is scored from 0 to 10 points. Each of the other scored domains consist of a series of multiple-choice questions with 5 selections per score from 1 to 5 points. The maximum score (100 points) indicates optimal state of the shoulder. Self-administered.	The physical scale can be found in [203].
25. SIMPLE SHOULDER TEST (SST) (Lippitt et al., 1993) [206]	Beaton et al., 1996 [20].	Beaton et al., 1998 [21], Roddey et al., 2000 [169], Cook et al., 2001 [133], Godfrey et al., 2007 [207], Oh et al., 2009 [24], Roy et al., 2010 [208], Hsu et al., 2017 [209], Vascellari et al., 2018 [96], Baumgarten et al., 2020 [31].	Shoulder pain [21], shoulder disorders [169], shoulder instability and RC injuries [207], shoulder arthroplasty [209], anterior shoulder instability surgery [96], proximal humerus fracture [210].	United States English [206], Italian [99], Dutch [211], Persian [212], Portuguese-Brazilian [213], Lithuanian [214], Spanish [215].	It is composed of 12 questions related to function, pain, strength, and ROM. The questions are on a dichotomous scale (1 = yes and 0 = no). The maximum score (12 points) indicates optimal state of the shoulder. Self-administered.	The physical scale can be found in [206].
26. SINGLE ASSESSMENT NUMERIC EVALUATION RATING (SANE) (Williams et al., 1999) [216]	Sciascia et al., 2017 [26].	Gowd et al., 2019 [217], Thigpen et al., 2018 [218], Cohn et al., 2020 [219].	Shoulder surgery [216], total shoulder arthroplasty [26,217,220], RC disease [221], glenoid labral pathology [131], shoulder instability [222].	United States English [216].	It consists of a single question about function. It is valued from 0 to 100 points. The question is “How would you rate your shoulder’s function with 100 being normal?” The maximum score (100 points) indicates optimal state of the shoulder. Self-administered.	The physical scale can be found in [216].
27. SUBJECTIVE SHOULDER RATING SCALE (SSRS) (Kohn et al., 1992) [223]	Beaton et al., 1996 [20].	Kohn et al. 1997 [224], Beaton et al., 1998 [21].	Shoulder disorders [21,224], shoulder pain [20].	Germany English [224].	It is composed of 5 components: pain (35 points), ROM (35 points), instability (15 points), activity (10 points), and overhead work (5 points). The maximum score (100 points) indicates optimal state of the shoulder. Self-administered.	The physical scale can be found in [224].
28. UNIVERSITY OF CALIFORNIA—LOS ANGELES SHOULDER SCALE (UCLA) (Amstutz et al., 1981) [225]	Romeo et al., 1996 [89].	Roddey et al., 2000 [169].	Shoulder instability [89], shoulder disorders [169], adhesive capsulitis [226], calcifying tendinitis of the shoulder [227], proximal humeral fractures [228], RC repair [229].	United States English [225].	It is composed of 3 components: pain (10 points), function (10 points), and muscle power and ROM (10 points). The maximum score (30 points) indicates optimal state of the shoulder. Self-administered section and clinician assessment section.	There is also a Modified UCLA [95]. The physical scale can be found in [225].
29. UNITED KINGDOM SHOULDER DISABILITY QUESTIONNAIRE (UK-SDQ) (Croft et al., 1994) [230]	Croft et al., 1994 [230].	Paul et al., 2004 [61].	Shoulder pain [61,230].	United Kingdom English [230], Italian [231].	It contains 22 items about problems with daily living related to shoulder pain. The questions are on a dichotomous scale (1 = yes and 0 = no). The maximum score (22 points) indicates the highest degree of disability. Self-administered.	The physical scale can be found in [230].
30. WESTERN ONTARIO SHOULDER INSTABILITY INDEX (WOSI) (Kirkley et al., 1998) [232]	Kirkley et al., 1998 [232].	Oh et al., 2009 [24], Kemp et al., 2012 [25], Van der Linde et al., 2017 [105].	Shoulder instability [232], shoulder surgery [24], surgical correction of shoulder instability [25,233], posterior shoulder instability [234], SLAP lesion or recurrent anterior dislocation [235].	United States English [232], French [236,237], Danish [238], Dutch [239,240], German [241], Hebrew [242], Italian [243], Japanese [244], Swedish [245] Turkish [246], Arabic [247,248], Spanish [249].	It contains 21 items that assess 4 domains: physical symptoms (10 items), sport/recreation work (4 items), lifestyle (4 items), and emotions (3 items). Each item has a possible score from 0 to 100 (100 mm visual analogue scale) and these scores are added to give a total score from 0 to 2100 points. The maximum score (2100 points) indicates the highest degree of disability. The score can be reported as a percentage of normal by subtracting the total from 2100, dividing by 2100, and multiplying by 100. e.g.,: (2100 − total score/2100) x 100. Self-administered.	The physical scale can be found in [232].
31. WESTERN ONTARIO OSTEOARTHRITIS OF THE SHOULDER INDEX (WOOS) (Lo et al., 2001) [250]	Lo et al., 2001 [250].	Sciascia et al., 2017 [26].	Osteoarthritis of the shoulder [250,251], total shoulder arthroplasty [26,252], proximal humeral fracture [253].	United States English [250], Danish [254], Italian [255], Swedish [256], Chinese [257].	It is composed of 19 items representing 4 domains: 6 questions for pain and physical symptoms; 5 for sport, recreation, and work function; 5 for lifestyle function; and 3 for emotional function. Each item has a possible score from 0 to 100 (100 mm visual analogue scale) and these scores are added to give a total score from 0 to 1900 points. The maximum score (1900 points) indicates the highest degree of disability. The score can be reported as a percentage of normal by subtracting the total from 1900, dividing by 1900, and multiplying by 100. e.g.,: (1900 − total score/1900) x 100. Self-administered.	The physical scale can be found in [250].
32. WESTERN ONTARIO ROTATOR CUFF INDEX (WORC) (Kirkley et al., 2003) [258]	Kirkley et al., 2003 [258].	Razmjou et al., 2006 [143], Gadsboell et al., 2017 [259].	RC disease [258,260], impingement syndrome, RC repair, acromioplasty, or decompression surgeries [143], scapula alata [259], subacromial impingement syndrome [261].	United States English [258], Brazilian-Portuguese [262], Chinese [263], Dutch [264,265], Japanese [266], Persian [267], Turkish [268], Danish [269], Canadian-French [270], Polish [271], Swedish [272].	It is composed of 21 items representing 5 domains: 6 questions in the physical symptoms domain, 4 in sports and recreation, 4 in work, 4 in lifestyle, and 3 in the emotional domain. Each item has a possible score from 0 to 100 (100 mm visual analogue scale) and these scores are added to give a total score from 0 to 2100 points. The maximum score (2100 points) indicates the highest degree of disability. The score can be reported as a percentage of normal by subtracting the total from 2100, dividing by 2100, and multiplying by 100. e.g.,: (2100 − total score/2100) x 100. Self-administered.	The physical scale can be found in [258].

Note: Some validation studies validated more than one shoulder tool. Some physical scales are found in papers that are not original or validation studies (see observations). The fourth column (applications/indications) shows a maximum of 6 applications or indications as examples.

**Table 3:** Assessment of the methodological quality with QUADAS-2.

		Risk of Bias			Applicability			
Tools	Validation Studies	Patient Selection	Index Test	Reference Standard	Flow and Timing	Patient Selection	Index Test	Reference Standard
1. AMERICAN SHOULDER AND ELBOW SURGEONS STANDARDIZED SHOULDER ASSESSMENT FORM (ASES) [19]	Beaton et al., 1996 [20] *	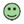	?	?	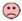	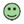	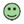	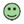
	Beaton et al., 1998 [21]	?	-	-	-	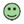	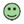	-
	Cook et al., 2002 [22]	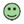	-	-	-	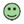	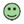	-
	Michener et al., 2002 [23]	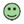	?	?	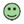	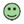	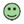	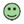
	Oh et al., 2009 [24]	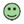	?	?	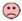	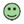	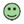	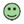
	Kemp et al., 2012 [25]	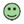	-	-	-	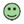	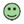	-
	Sciascia et al., 2017 [26]	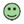	?	?	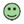	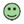	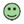	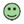
	Dabija et al., 2019 [27]	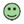	?	?	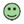	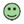	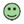	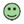
	Vrotsou et al., 2019 [28]	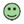	-	-	-	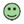	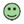	-
	Gotlin et al., 2020 [29]	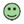	-	-	-	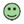	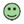	-
	Hou et al., 2020 [30]	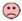	-	-	-	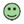	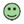	-
	Baumgarten et al., 2020 [31]	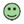	-	-	-	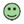	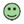	-
2. CONSTANT-MURLEY SCORE (CMS) [44]	Conboy et al., 1996 [45] *	?	-	-	-	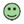	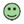	-
	Cook et al., 2002 [22]	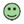	-	-	-	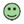	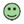	-
	Angst et al., 2008 [46]	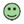	-	-	-	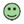	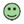	-
	Razmjou et al., 2008 [47]	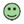	?	?	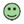	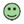	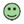	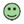
	Rocourt et al., 2008 [48]	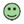	-	-	-	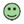	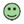	-
	Oh et al., 2009 [24]	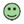	?	?	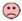	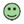	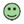	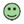
	Kemp et al., 2012 [25]	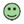	-	-	-	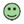	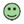	-
	Ban et al., 2016 [49]	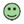	?	?	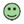	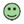	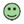	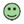
	Mahabier et al., 2016 [50]	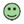	?	?	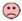	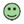	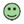	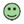
	Sciascia et al., 2017 [26]	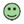	?	?	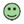	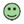	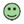	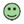
	James-Belin et al., 2018 [51]	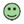	-	-	-	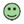	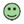	-
3. DUTCH SHOULDER DISABILITY QUESTIONNAIRE (DUTCH-SDQ) [58]	Van der Windt et al., 1998 [59] *	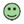	?	?	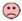	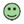	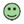	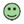
	Van der Heijden et al., 2000 [60]	?	-	-	-	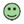	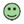	-
	Paul et al., 2004 [61]	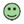	?	?	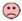	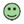	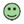	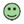
4. FLEXILEVEL SCALE OF SHOULDER FUNCTION (FLEX-SF) [63]	Cook et al., 2003 [63] *	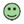	?	?	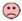	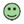	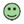	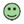
5. FUDAN UNIVERSITY SHOULDER SCORE [70]	Ge et al., 2013 [70] *	?	?	?	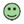	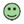	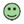	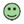
6. FUNCTIONAL SHOULDER SCORE (FSS) [72]	Iossifidis et al., 2015 [72] *	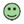	?	?	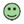	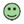	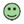	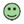
7. KOREAN SHOULDER SCORING SYSTEM (KSS) [73]	Tae et al., 2009 [73] *	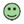	?	?	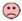	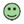	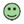	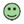
8. MELBOURNE INSTABILITY SHOULDER SCALE (MISS) [80]	Watson et al., 2005 [80] *	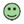	-	-	-	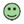	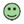	-
9. MODIFIED CONSTANT-MURLEY SCORE [82]	Van der Water et al., 2014 [83] *	?	?	?	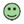	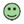	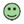	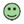
10. MODIFIED ROWE SHOULDER SCORE (MRS) [88]	Romeo et al., 1996 [89] *	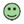	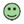	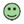	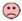	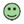	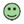	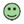
11. MODIFIED UNIVERSITY OF CALIFORNIA—LOS ANGELES SHOULDER SCALE (UCLA) [95]	Cook et al., 2002 [22] *	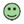	-	-	-	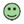	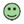	-
	Oh et al., 2009 [24]	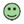	?	?	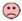	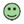	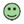	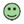
	Van de Water et al., 2014 [83]	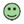	?	?	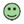	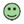	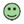	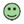
	Vascellari et al., 2018 [96]	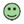	?	?	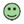	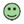	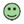	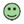
12. MUNICH SHOULDER QUESTIONNAIRE (MSQ) [100]	Schmidutz et al., 2012 [100] *	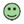	?	?	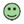	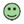	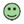	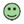
13. OXFORD INSTABILITY SCORE (OIS) [104]	Dawson et al., 1999 [104] *	?	?	?	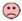	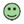	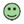	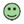
	Van der Linde et al., 2017 [105]	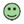	?	?	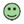	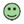	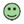	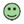
14. OXFORD SHOULDER SCORE (OSS) [111]	Dawson et al., 1996 [111] *	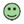	?	?	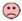	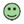	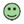	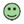
	Van de Water et al., 2014 [83]	?	?	?	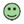	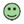	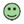	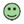
15. PEDIATRIC/ ADOLESCENT SHOULDER SURVEY (PASS) [129]	Edmonds et al., 2017 [129] *	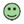	?	?	?	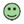	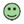	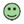
16. PENN SHOULDER SCORE (PSS) [132]	Cook et al., 2001 [133] *	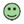	-	-	-	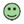	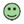	-
	Leggin et al., 2006 [134]	?	?	?	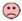	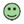	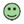	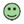
17. ROTATOR CUFF QUALITY OF LIFE (RC-QOL) [142]	Hollinshead et al., 2000 [142] *	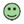	?	?	?	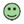	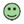	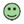
	Razmjou et al., 2006 [143]	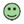	?	?	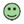	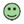	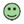	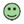
	Eubank et al., 2017 [144]	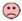	?	?	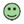	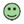	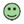	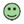
18. ROWE SCALE [94]	Romeo et al., 1996 [89] *	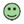	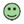	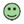	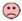	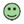	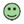	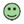
	Oh et al., 2009 [24]	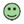	?	?	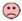	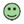	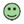	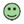
19. SHORT WESTERN ONTARIO ROTATOR CUFF INDEX (SHORT-WORC) [159]	Razmjou et al., 2012 [159] *	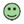	?	?	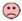	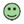	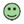	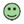
	Dewan et al., 2016 [160]	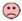	?	?	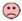	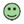	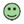	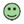
	Dewan et al., 2018 [161]	?	?	?	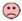	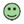	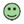	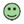
	Furtado et al., 2020 [162]	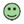	-	-	-	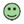	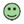	-
20. SHOULDER ACTIVITY RATING SCALE (SARS) [12]	Brophy et al., 2005 [12] *	?	?	?	?	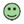	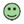	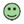
21. SHOULDER FUNCTION INDEX (SFInX) [165]	Van de Water et al., 2015 [165] *	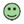	-	-	-	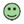	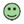	-
	Van de Water et al., 2015 [166]	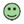	?	?	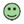	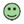	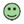	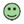
22. SHOULDER PAIN AND DISABILITY INDEX (SPADI) [167]	Roach et al., 1991 [167] *	?	?	?	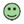	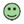	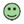	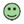
	Beaton et al., 1996 [20]	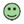	?	?	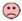	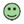	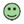	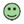
	Heald et al., 1997 [168]	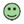	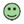	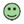	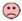	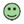	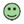	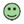
	Beaton et al., 1998 [21]	?	-	-	-	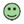	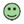	-
	Roddey et al., 2000 [169]	?	?	?	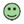	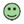	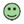	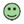
	Cook et al., 2001 [133]	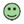	-	-	-	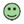	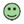	-
	Cook et al., 2002 [22]	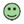	-	-	-	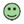	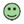	-
	Paul et al., 2004 [61]	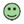	?	?	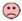	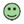	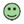	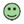
	MacDermid et al., 2006 [170]	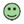	?	?	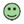	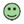	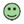	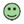
	Angst et al., 2008 [46]	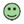	-	-	-	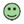	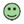	-
	Bicer et al., 2010 [171]	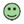	?	?	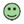	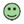	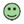	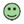
	Staples et al., 2010 [172]	?	?	?	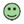	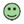	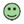	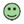
	Hill et al., 2011 [173]	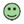	?	?	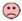	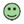	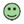	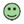
	Riley et al., 2015 [174]	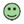	-	-	-	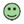	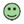	-
	Jerosch-Herold et al., 2017 [175]	?	-	-	-	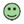	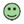	-
	Thoomes de Graaf et al., 2017 [176]	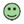	?	?	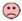	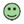	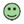	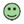
	James-Berlin et al., 2018 [51]	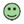	-	-	-	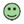	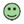	-
	Vascellari et al., 2018 [96]	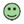	?	?	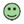	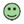	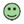	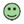
	Riley et al., 2019 [177]	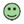	-	-	-	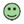	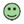	-
	Dabija et al., 2019 [27]	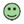	?	?	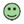	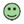	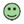	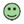
	Boake et al., 2020 [178]	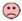	-	-	-	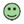	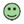	-
23. SHOULDER PAIN SCORE (SPS) [196]	Winters et al., 1996 [196] *	?	-	-	-	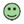	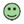	-
24. SHOULDER RATING QUESTIONNAIRE (SRQ) [203]	L’Insalata et al., 1997 [203] *	?	?	?	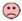	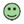	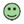	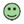
	Paul et al., 2004 [61]	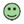	?	?	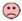	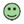	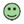	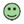
25. SIMPLE SHOULDER TEST (SST) [206]	Beaton et al., 1996 [20] *	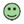	?	?	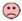	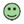	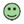	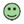
	Beaton et al., 1998 [21]	?	-	-	-	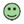	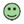	-
	Roddey et al., 2000 [169]	?	?	?	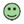	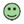	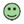	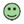
	Cook et al., 2001 [133]	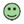	-	-	-	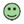	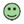	-
	Godfrey et al., 2007 [207]	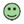	?	?	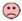	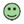	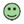	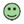
	Oh et al., 2009 [24]	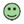	?	?	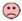	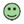	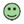	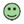
	Roy et al., 2010 [208]	?	?	?	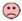	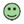	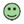	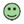
	Hsu et al., 2017 [209]	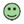	?	?	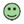	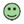	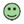	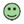
	Vascellari et al., 2018 [96]	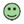	?	?	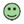	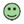	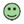	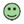
	Baumgarten et al., 2020 [31]	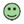	-	-	-	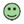	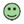	-
26. SINGLE ASSESSMENT NUMERIC EVALUATION RATING (SANE) [216]	Sciascia et al., 2017 [26] *	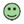	?	?	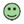	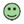	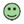	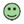
	Gowd et al., 2019 [217]	?	-	-	-	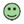	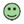	-
	Thigpen et al., 2018 [218]	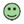	?	?	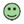	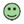	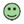	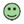
	Cohn et al., 2020 [219]	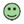	?	?	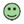	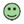	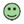	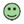
27. SUBJECTIVE SHOULDER RATING SCALE (SSRS) [223]	Beaton et al., 1996 [20] *	?	?	?	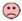	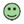	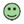	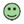
	Kohn et al., 1997 [224]	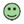	?	?	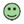	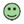	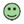	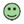
	Beaton et al., 1998 [21]	?	-	-	-	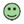	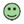	-
28. UNIVERSITY OF CALIFORNIA—LOS ANGELES SHOULDER SCALE (UCLA) [225]	Romeo et al., 1996 [89] *	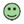	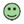	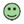	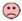	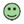	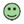	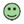
	Roddey et al., 2000 [169]	?	?	?	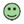	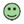	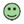	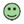
29. UNITED KINGDOM SHOULDER DISABILITY QUESTIONNAIRE (UK-SDQ) [230]	Croft et al., 1994 [230] *	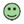	-	-	-	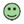	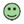	-
	Paul et al., 2004 [61]	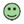	?	?	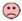	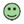	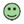	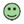
30. WESTERN ONTARIO SHOULDER INSTABILITY INDEX (WOSI) [232]	Kirkley et al., 1998 [232] *	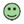	?	?	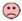	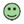	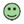	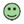
	Oh et al., 2009 [24]	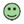	?	?	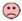	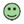	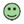	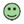
	Kemp et al., 2012 [25]	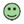	-	-	-	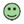	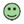	-
	Van der Linde et al., 2017 [105]	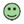	?	?	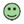	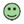	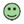	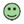
31. WESTERN ONTARIO OSTEOARTHRITIS OF THE SHOULDER INDEX (WOOS) [250]	Lo et al., 2001 [250] *	?	?	?	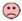	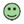	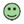	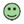
	Sciascia et al., 2017 [26]	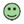	?	?	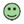	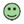	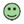	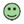
32. WESTERN ONTARIO ROTATOR CUFF INDEX (WORC) [258]	Kirkley et al., 2003 [258] *	?	?	?	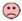	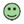	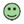	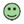
	Razmjou et al., 2006 [143]	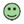	?	?	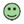	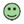	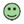	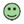
	Gadsboell et al., 2017 [259]	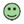	-	-	-	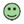	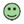	-

Note: * Original validation studies. There were validation studies which validated more than one shoulder tool. Interpretation: 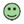, low risk of bias or low concerns regarding applicability; 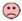, high risk of bias or high concerns regarding applicability; ?, unclear risk of bias or unclear concerns regarding applicability; -, not applicable.

**Table 4:** Assessment of methodological quality with COSMIN Risk of Bias checklist.

Tools	Validation Studies	PROMs Development	Content Validity	Structural Validity	Internal Consistency	Reliability	MEASUREMENT ERROR	Criterion Validity	Construct Validity	Responsiveness
1. AMERICAN SHOULDER AND ELBOW SURGEONS STANDARDIZED SHOULDER ASSESSMENT FORM (ASES) [19]	Beaton et al., 1996 [20] *								Very good	
	Beaton et al., 1998 [21]					Adequate				Doubtful
	Cook et al., 2002 [22]				Very good	Inadequate				
	Michener et al., 2002 [23]				Very good	Adequate	Inadequate		Very good	Inadequate
	Oh et al., 2009 [24]				Very good			Very good	Very good	Doubtful
	Kemp et al., 2012 [25]								Adequate	Very good
	Sciascia et al., 2017 [26]									Inadequate
	Dabija et al., 2019 [27]					Inadequate			Inadequate	Doubtful
	Vrotsou et al., 2019 [28]					Inadequate				
	Gotlin et al., 2020 [29]					Inadequate				
	Hou et al., 2020 [30]					Doubtful				
	Baumgarten et al., 2020 [31]									Doubtful
2. CONSTANT-MURLEY SCORE (CMS) [44]	Conboy et al., 1996 [45] *					Inadequate	Adequate			
	Cook et al., 2002 [22]					Inadequate				
	Angst et al., 2008 [46]									Doubtful
	Razmjou et al., 2008 [47]				Very good				Inadequate	
	Rocourt et al., 2008 [48]					Adequate				
	Oh et al., 2009 [24]				Inadequate			Doubtful	Very good	Doubtful
	Kemp et al., 2012 [25]								Adequate	Very good
	Ban et al., 2016 [49]				Inadequate	Adequate	Adequate		Very good	
	Mahabier et al., 2016 [50]				Very good				Very good	Very good
	Sciascia et al., 2017 [26]									Inadequate
	James-Belin et al., 2018 [51]					Adequate				Very good
3. DUTCH SHOULDER DISABILITY QUESTIONNAIRE (DUTCH-SDQ) [58]	Van der Windt et al., 1998 [59] *									Very good
	Van der Heijden et al., 2000 [60]	Inadequate								Very good
	Paul et al., 2004 [61]							Very good		Inadequate
4. FLEXILEVEL SCALE OF SHOULDER FUNCTION (FLEX-SF) [63]	Cook et al., 2003 [63] *	Inadequate				Inadequate			Inadequate	Inadequate
5. FUDAN UNIVERSITY SHOULDER SCORE [70]	Ge et al., 2013 [70] *	Doubtful				Very good			Inadequate	Inadequate
6. FUNCTIONAL SHOULDER SCORE (FSS) [72]	Iossifidis et al., 2015 [72] *	Inadequate		Adequate	Very good	Inadequate	Very good		Very good	Doubtful
7. KOREAN SHOULDER SCORING SYSTEM (KSS) [73]	Tae et al., 2009 [73] *	Inadequate	Inadequate		Very good			Very good	Inadequate	Doubtful
8. MELBOURNE INSTABILITY SHOULDER SCALE (MISS) [80]	Watson et al., 2005 [80] *	Inadequate				Adequate	Adequate			
9. MODIFIED CONSTANT-MURLEY SCORE [82]	Van der Water et al., 2014 [83] *		Doubtful			Adequate	Adequate		Very good	Very good
10. MODIFIED ROWE SHOULDER SCORE (MRS) [88]	Romeo et al., 1996 [89] *					Inadequate				
11. MODIFIED UNIVERSITY OF CALIFORNIA—LOS ANGELES SHOULDER SCALE (UCLA) [95]	Cook et al., 2002 [22] *					Inadequate				
	Oh et al., 2009 [24]				Inadequate			Doubtful	Very good	Doubtful
	Van de Water et al., 2014 [83]		Doubtful			Adequate	Adequate		Very good	Very good
	Vascellari et al., 2018 [96]					Adequate	Adequate		Very good	
12. MUNICH SHOULDER QUESTIONNAIRE (MSQ) [100]	Schmidutz et al., 2012 [100] *	Inadequate							Inadequate	
13. OXFORD INSTABILITY SCORE (OIS) [104]	Dawson et al., 1999 [104] *	Inadequate			Very good	Doubtful			Inadequate	Inadequate
	Van der Linde et al., 2017 [105]									Inadequate
14. OXFORD SHOULDER SCORE (OSS) [111]	Dawson et al., 1996 [111] *	Inadequate			Doubtful	Doubtful			Adequate	Doubtful
	Van de Water et al., 2014 [83]		Doubtful			Adequate	Adequate		Very good	Very good
15. PEDIATRIC/ADOLESCENT SHOULDER SURVEY (PASS) [129]	Edmonds et al., 2017 [129] *	Inadequate			Very good	Doubtful			Very good	Doubtful
16. PENN SHOULDER SCORE (PSS) [132]	Cook et al., 2001 [133] *					Inadequate				
	Leggin et al., 2006 [134]				Inadequate	Doubtful	Inadequate		Inadequate	Inadequate
17. ROTATOR CUFF QUALITY OF LIFE (RC-QOL) [142]	Hollinshead et al., 2000 [142] *	Doubtful				Inadequate			Doubtful	
	Razmjou et al., 2006 [143]								Very good	Doubtful
	Eubank et al., 2017 [144]		Doubtful		Very good	Doubtful			Very good	Inadequate
18. ROWE SCALE [94]	Romeo et al., 1996 [89] *					Inadequate				
	Oh et al., 2009 [24]				Inadequate			Very good	Very good	Doubtful
19. SHORT WESTERN ONTARIO ROTATOR CUFF INDEX (SHORTWORC) [159]	Razmjou et al., 2012 [159] *	Inadequate			Very good		Doubtful		Inadequate	Inadequate
	Dewan et al., 2016 [160]				Very good	Adequate	Adequate			
	Dewan et al., 2018 [161]								Inadequate	Inadequate
	Furtado et al., 2020 [162]		Adequate							
20. SHOULDER ACTIVITY RATING SCALE (SARS) [12]	Brophy et al., 2005 [12] *	Doubtful				Doubtful			Inadequate	
21. SHOULDER FUNCTION INDEX (SFInX) [165]	Van de Water et al., 2015 [165] *	Inadequate								
	Van de Water et al., 2015 [166]					Adequate	Adequate		Adequate	Adequate
22. SHOULDER PAIN AND DISABILITY INDEX (SPADI) [167]	Roach et al., 1991 [167] *	Inadequate			Doubtful	Inadequate		Very good	Inadequate	
	Beaton et al., 1996 [20]								Very good	
	Heald et al., 1997 [168]								Very good	Inadequate
	Beaton et al., 1998 [21]					Adequate				Doubtful
	Roddey et al., 2000 [169]				Very good	Inadequate			Very good	
	Cook et al., 2001 [133]					Inadequate				
	Cook et al., 2002 [22]				Very good	Inadequate				
	Paul et al., 2004 [61]							Very good		Inadequate
	MacDermid et al., 2006 [170]			Inadequate	Very good				Very good	Very good
	Angst et al., 2008 [46]									Doubtful
	Bicer et al., 2010 [171]				Very good	Adequate			Very good	
	Staples et al., 2010 [172]								Very good	Doubtful
	Hill et al., 2011 [173]			Inadequate	Very good				Very good	
	Riley et al., 2015 [174]									Very good
	Jerosch-Herold et al., 2017 [175]			Very good						
	Thoomes de Graaf et al., 2017 [176]					Adequate	Adequate			Adequate
	James-Berlin et al., 2018 [51]					Adequate				Very good
	Vascellari et al., 2018 [96]				Very good	Adequate	Adequate		Very good	
	Riley et al., 2019 [177]					Doubtful				
	Dabija et al., 2019 [27]					Inadequate			Inadequate	Doubtful
	Boake et al., 2020 [178]			Very good		Inadequate				
23. SHOULDER PAIN SCORE (SPS) [196]	Winters et al., 1996 [196] *	Inadequate		Doubtful	Very good					
24. SHOULDER RATING QUESTIONNAIRE (SRQ) [203]	L’Insalata et al., 1997 [203] *	Inadequate			Very good	Doubtful		Doubtful	Adequate	Inadequate
	Paul et al., 2004 [61]							Very good		Very good
25. SIMPLE SHOULDER TEST (SST) [206]	Beaton et al., 1996 [20] *								Very good	
	Beaton et al., 1998 [21]					Adequate				Doubtful
	Roddey et al., 2000 [169]				Very good	Inadequate			Very good	
	Cook et al., 2001 [133]					Inadequate				
	Godfrey et al., 2007 [207]		Inadequate			Adequate		Very good	Very good	
	Oh et al., 2009 [24]				Very good			Doubtful	Very good	Doubtful
	Roy et al., 2010 [208]								Very good	Doubtful
	Hsu et al., 2017 [209]		Inadequate					Very good		Doubtful
	Vascellari et al., 2018 [96]				Very good	Adequate	Adequate		Very good	
	Baumgarten et al., 2020 [31]									Doubtful
26. SINGLE ASSESSMENT NUMERIC EVALUATION RATING (SANE) [216]	Sciascia et al., 2017 [26] *									Inadequate
	Gowd et al., 2019 [217]									Inadequate
	Thigpen et al., 2018 [218]					Adequate	Adequate	Very good	Inadequate	Doubtful
	Cohn et al., 2020 [219]								Inadequate	
27. SUBJECTIVE SHOULDER RATING SCALE (SSRS) [223]	Beaton et al., 1996 [20] *								Very good	
	Kohn et al.1997 [224]	Inadequate				Inadequate				
	Beaton et al., 1998 [21]					Adequate				Doubtful
28. UNIVERSITY OF CALIFORNIA—LOS ANGELES SHOULDER SCALE (UCLA) [225]	Romeo et al., 1996 [89] *					Inadequate				
	Roddey et al., 2000 [169]								Very good	
29. UNITED KINGDOM SHOULDER DISABILITY QUESTIONNAIRE (UK-SDQ) [230]	Croft et al., 1994 [230] *	Inadequate	Inadequate							
	Paul et al., 2004 [61]							Very good		Inadequate
30. WESTERN ONTARIO SHOULDER INSTABILITY INDEX (WOSI) [232]	Kirkley et al., 1998 [232] *	Doubtful				Inadequate			Inadequate	Inadequate
	Oh et al., 2009 [24]				Very good			Very good	Very good	Doubtful
	Kemp et al., 2012 [25]								Adequate	Very good
	Van der Linde et al., 2017 [105]									Inadequate
31. WESTERN ONTARIO OSTEOARTHRITIS OF THE SHOULDER INDEX (WOOS) [250]	Lo et al., 2001 [250] *	Doubtful				Adequate			Inadequate	Doubtful
	Sciascia et al., 2017 [26]									Inadequate
32. WESTERN ONTARIO ROTATOR CUFF INDEX (WORC) [258]	Kirkley et al., 2003 [258] *	Doubtful				Adequate			Inadequate	
	Razmjou et al., 2006 [143]								Very good	Doubtful
	Gadsboell et al., 2017 [259]		Inadequate							

Note: * Original validation studies. There were some validation studies which validated more than one shoulder tool. Only one section was supressed (cross-cultural validity/measurement invariance), as it was not considered in any of the validated articles included. Abbreviations: PROMs, patient reported outcome measures.

**Table 5:** Study population of the validations.

Tools	Validation Studies	Populations		
		Symptoms/ Signs	Pathologies	Surgical Interventions
1. AMERICAN SHOULDER AND ELBOW SURGEONS STANDARDIZED SHOULDER ASSESSMENT FORM (ASES) [19]	Beaton et al., 1996 [20] *		Shoulder disorders	
	Beaton et al., 1998 [21]	Pain	RC disease, OA, glenohumeral instability, malunion of a shoulder fx	RC repair, total shoulder arthroplasty
	Cook et al., 2002 [22]	Shoulder dysfunction		
	Michener et al., 2002 [23]	Weakness	Impingement syndrome, instability/dislocation, RC syndrome, adhesive capsulitis, humeral fx, RC and adhesive capsulitis	Surgery
	Oh et al., 2009 [24]		RC disorder, SLAP lesion, shoulder instability	
	Kemp et al., 2012 [25]	Symptoms of shoulder instability	Shoulder instability	
	Sciascia et al., 2017 [26]		Primary glenohumeral OA	Total shoulder arthroplasty
	Dabija et al., 2019 [27]		RC tear	
	Vrotsou et al., 2019 [28]		Subacromial pathology with/without RC rupture, tendinopathy, instability	Surgery repair
	Gotlin et al., 2020 [29]			RC repair
	Hou et al., 2020 [30]	Pain	RC tear, frozen shoulder, impingement syndrome, instability of shoulder, AC joint arthritis, SLAP lesion, biceps tendinopathy	
	Baumgarten et al., 2020 [31]			RC repair and total shoulder arthroplasty
2. CONSTANT-MURLEY SCORE (CMS) [44]	Conboy et al., 1996 [45] *		Dislocation, arthritis, impingement	
	Cook et al., 2002 [22]	Shoulder dysfunction		
	Angst et al., 2008 [46]			Primary unilateral or bilateral total shoulder arthroplasty
	Razmjou et al., 2008 [47]		Impingement syndrome or partial thickness RC tears	RC repair
	Rocourt et al., 2008 [48]	Shoulder dysfunctions		
	Oh et al., 2009 [24]		RC disorders, isolated SLAP lesions, shoulder instability	
	Kemp et al., 2012 [25]	Anterior glenohumeral instability	Shoulder instability	
	Ban et al., 2016 [49]		Clavicle fx	
	Mahabier et al., 2016 [50]		Humeral shaft fx	
	Sciascia et al., 2017 [26]		Glenohumeral OA	Total shoulder arthroplasty
	James-Belin et al., 2018 [51]	Pain	Degenerative RC disease (tendinopathy with or without full-thickness tear)	
3. DUTCH SHOULDER DISABILITY QUESTIONNAIRE (DUTCH-SDQ) [58]	Van der Windt et al., 1998 [59] *	Pain	Capsular syndrome, acute bursitis, acromioclavicular syndrome, subacromial syndrome	
	Van der Heijden et al., 2000 [60]	Pain and restricted passive ROM glenohumeral		
	Paul et al., 2004 [61]	Pain		
4. FLEXILEVEL SCALE OF SHOULDER FUNCTION (FLEX-SF) [63]	Cook et al., 2003 [63] *		Shoulder pathology	
5. FUDAN UNIVERSITY SHOULDER SCORE [70]	Ge et al., 2013 [70] *	Pain or discomfort	RC tear, biceps tendon injury, subacromial impingement, labrum injury, frozen shoulder, tendinopathy	
6. FUNCTIONAL SHOULDER SCORE (FSS) [72]	Iossifidis et al., 2015 [72] *		RC disease	RC repair
7. KOREAN SHOULDER SCORING SYSTEM (KSS) [73]	Tae et al., 2009 [73] *		RC tears, impingement syndrome or RC tendinopathy	
8. MELBOURNE INSTABILITY SHOULDER SCALE (MISS) [80]	Watson et al., 2005 [80] *		Glenohumeral dislocation or subluxation	Surgical stabilization
9. MODIFIED CONSTANT-MURLEY SCORE [82]	Van der Water et al., 2014 [83] *		Isolated proximal humeral fx	
10. MODIFIED ROWE SHOULDER SCORE (MRS) [88]	Romeo et al., 1996 [89] *		Shoulder stabilization procedures	Bankart-type repairs, capsular shifts, arthroscopic stabilizations
11. MODIFIED UNIVERSITY OF CALIFORNIA—LOS ANGELES SHOULDER SCALE (UCLA) [95]	Cook et al., 2002 [22] *		Shoulder dysfunction	
	Oh et al., 2009 [24]		RC disorders, isolated SLAP lesions, shoulder instability	
	Van de Water et al., 2014 [83]		Isolated proximal humeral fx	
	Vascellari et al., 2018 [96]		Anterior shoulder instability	Arthroscopic Bankart repair, open Bristow-Latarjet procedure **
12. MUNICH SHOULDER QUESTIONNAIRE (MSQ) [100]	Schmidutz et al., 2012 [100] *		Shoulder disorder	
13. OXFORD INSTABILITY SCORE (OIS) [104]	Dawson et al., 1999 [104] *		Shoulder instability	
	Van der Linde et al., 2017 [105]		Primary and recurrent shoulder instability	
14. OXFORD SHOULDER SCORE (OSS) [111]	Dawson et al., 1996 [111] *		Impingement syndrome, RC tear, calcified deposits in the RC tendon, primary or secondary OA, inflammatory arthritis, adhesive capsulitis	
	Van de Water et al., 2014 [83]		Isolated proximal humeral fracture	
15. PEDIATRIC/ ADOLESCENT SHOULDER SURVEY (PASS) [129]	Edmonds et al., 2017 [129] *	Complaints related to the shoulder		
16. PENN SHOULDER SCORE (PSS) [132]	Cook et al., 2001 [133] *	Pain and dysfunction		
	Leggin et al., 2006 [134]		Impingement/tendinopathy, RC tear, instability, adhesive capsulitis/frozen shoulder, proximal humerus fx, acromioclavicular joint arthritis, glenohumeral joint arthritis	
17. ROTATOR CUFF QUALITY OF LIFE (RC-QOL) [142]	Hollinshead et al., 2000 [142] *		RC disease	
	Razmjou et al., 2006 [143]		Impingement syndrome	RC repair
	Eubank et al., 2017 [144]		Chronic full-thickness RC tear	
18. ROWE SCALE [94]	Romeo et al., 1996 [89] *		Shoulder stabilization procedures	Bankart-type repairs, capsular shifts, arthroscopic stabilizations
	Oh et al., 2009 [24]		RC disorders, isolated SLAP lesions, shoulder instability	
19. SHORT WESTERN ONTARIO ROTATOR CUFF INDEX (SHORT-WORC) [159]	Razmjou et al., 2012 [159] *		RC pathology with biceps lesion	Acromioplasty, RC repair, debridement, tenodesis or tenotomy of LHB
	Dewan et al., 2016 [160]			RC repair with or without acromioplasty
	Dewan et al., 2018 [161]			RC repair
	Furtado et al., 2020 [162]		RC disorders	
20. SHOULDER ACTIVITY RATING SCALE (SARS) [12]	Brophy et al., 2005 [12] *		RC tears, glenohumeral joint OA, RC arthropathy	Reverse total shoulder arthroplasty
21. SHOULDER FUNCTION INDEX (SFInX) [165]	Van de Water et al., 2015 [165] *		Isolated proximal humeral fx, proximal humeral fx-dislocation	
	Van de Water et al., 2015 [166]		Isolated proximal humeral fx or proximal humeral fx-dislocation	
22. SHOULDER PAIN AND DISABILITY INDEX (SPADI) [167]	Roach et al., 1991 [167] *	Pain		
	Beaton et al., 1996 [20]		Shoulder disorders	
	Heald et al., 1997 [168]	Pain, weakness	Impingement/tendinopathy/ bursitis, instability/dislocation, RC syndrome, adhesive capsulitis, fx, sternoclavicular or acromioclavicular joint subluxation, contusion	Arthroscopic surgery, RC repair
	Beaton et al., 1998 [21]	Pain	RC disease, OA, glenohumeral instability, malunion of a shoulder fx	RC repair, total shoulder arthroplasty
	Roddey et al., 2000 [169]		Shoulder disorders	
	Cook et al., 2001 [133]	Pain and dysfunction		
	Cook et al., 2002 [22]	Dysfunction		
	Paul et al., 2004 [61]	Pain		
	MacDermid et al., 2006 [170]	Pain		
	Angst et al., 2008 [46]			Primary unilateral or bilateral total shoulder arthroplasty
	Bicer et al., 2010 [171]	Pain	Adhesive capsulitis, RC/biceps tendinopathy, RC tear, myofascial, OA, bursitis	
	Staples et al., 2010 [172]	Pain, stiffness	Adhesive capsulitis	
	Hill et al., 2011 [173]	Pain, stiffness		
	Riley et al., 2015 [174]	Pain		
	Jerosch-Herold et al., 2017 [175]	Pain		
	Thoomes de Graaf et al., 2017 [176]	Pain		
	James-Berlin et al., 2018 [51]	Pain	Degenerative RC disease (tendinopathy with or without full-thickness tear)	
	Vascellari et al., 2018 [96]		Anterior shoulder instability	Arthroscopic Bankart repair, open Bristow-Latarjet procedure **
	Riley et al., 2019 [177]	Pain		
	Dabija et al., 2019 [27]		RC tears	
	Boake et al., 2020 [178]			RC repair
23. SHOULDER PAIN SCORE (SPS) [196]	Winters et al., 1996 [196] *	Pain		
24. SHOULDER RATING QUESTIONNAIRE (SRQ) [203]	L’Insalata et al., 1997 [203] *		Impingement instability, complete tear of the RC, OA of the glenohumeral joint, adhesive capsulitis, OA of the acromioclavicular joint	
	Paul et al., 2004 [61]	Pain		
25. SIMPLE SHOULDER TEST (SST) [206]	Beaton et al., 1996 [20] *		Shoulder disorder	
	Beaton et al., 1998 [21]	Pain	RC disease, OA, glenohumeral instability, malunion of a shoulder fx	RC repair, total shoulder arthroplasty
	Roddey et al., 2000 [169]		Shoulder disorders	
	Cook et al., 2001 [133]	Pain and dysfunction		
	Godfrey et al., 2007 [207]		Shoulder instability, RC injury	
	Oh et al., 2009 [24]		RC disorders, isolated SLAP lesions, shoulder instability	
	Roy et al., 2010 [208]			Shoulder arthroplasty: hemiarthroplasty, total shoulder arthroplasty, reverse total shoulder arthroplasty
	Hsu et al., 2017 [209]		OA, rheumatoid arthritis, avascular necrosis, capsulorrhaphy arthropathy, post-traumatic arthritis, cuff tear arthropathy	Shoulder arthroplasty
	Vascellari et al., 2018 [96]		Anterior shoulder instability	Arthroscopic Bankart repair, open Bristow-Latarjet procedure **
	Baumgarten et al., 2020 [31]			RC repair and total shoulder arthroplasty
26. SINGLE ASSESSMENT NUMERIC EVALUATION RATING (SANE) [216]	Sciascia et al., 2017 [26] *		Primary glenohumeral OA	Total shoulder arthroplasty
	Gowd et al., 2019 [217]		Primary glenohumeral arthritis and RC arthropathy	Anatomic or reverse total shoulder arthroplasty
	Thigpen et al., 2018 [218]	Signs and symptoms of subacromial impingement or adhesive capsulitis		Primary arthroscopic RC repair, total shoulder replacement
	Cohn et al., 2020 [219]			Total shoulder arthroplasty or reverse total shoulder arthroplasty
27. SUBJECTIVE SHOULDER RATING SCALE (SSRS) [223]	Beaton et al., 1996 [20] *		Shoulder disorders	
	Kohn et al., 1997 [224]			Anterior shoulder reconstructions, subacromial decompressions
	Beaton et al., 1998 [21]	Pain	RC disease, OA, glenohumeral instability, malunion of a shoulder fx	RC repair, total shoulder arthroplasty
28. UNIVERSITY OF CALIFORNIA—LOS ANGELES SHOULDER SCALE (UCLA) [225]	Romeo et al., 1996 [89] *		Shoulder stabilization procedures	Bankart-type repairs, capsular shifts, arthroscopic stabilizations
	Roddey et al., 2000 [169]		Shoulder disorders	
29. UNITED KINGDOM SHOULDER DISABILITY QUESTIONNAIRE (UK-SDQ) [230]	Croft et al., 1994 [230] *	Pain		
	Paul et al., 2004 [61]	Pain		
30. WESTERN ONTARIO SHOULDER INSTABILITY INDEX (WOSI) [232]	Kirkley et al., 1998 [232] *		Instability shoulder	
	Oh et al., 2009 [24]		RC disorder, SLAP lesion, shoulder instability	
	Kemp et al., 2012 [25]	Symptoms of shoulder instability	Shoulder instability	
	Van der Linde et al., 2017 [105]		Primary and recurrent shoulder instability	
31. WESTERN ONTARIO OSTEOARTHRITIS OF THE SHOULDER INDEX (WOOS) [250]	Lo et al., 2001 [250] *	Pain	OA	
	Sciascia et al., 2017 [26]		Primary glenohumeral OA	Total shoulder arthroplasty
32. WESTERN ONTARIO ROTATOR CUFF INDEX (WORC) [258]	Kirkley et al., 2003 [258] *	Symptoms	RC tendinopathy, RC tendinopathy with no tear, partial-thickness RC tears, full-thickness RC tears, RC arthropathy	
	Razmjou et al., 2006 [143]		Impingement syndrome	
	Gadsboell et al., 2017 [259]	Scapula alata		

Note: * Original validation studies. ** Specific surgical technique. Abbreviations: fx, fracture; OA, osteoarthritis; RC, rotator cuff; ROM, range of motion.

**Table 6:** Content approached by items and components of outcome measures.

Outcome Measures	ROM	Shoulder Stability	Pain	Patient/Clinician Satisfaction	Muscle Power/Strength	Physical Symptoms/Signs	ADL	Physical and Sport Activities	Work	Social Life	Psychological Aspects
1. AMERICAN SHOULDER AND ELBOW SURGEONS STANDARDIZED SHOULDER ASSESSMENT FORM (ASES) [19]	✓	✓	✓		✓	✓	✓				
2. CONSTANT-MURLEY SCORE (CMS) [44]	✓		✓		✓		✓	✓	✓		
3. DUTCH SHOULDER DISABILITY QUESTIONNAIRE (DUTCH-SDQ) [58]	✓		✓				✓				✓
4. FLEXILEVEL SCALE OF SHOULDER FUNCTION (FLEX-SF) [63]	✓				✓		✓		✓		
5. FUDAN UNIVERSITY SHOULDER SCORE [70]	✓		✓	✓	✓		✓				
6. FUNCTIONAL SHOULDER SCORE (FSS) [72]			✓				✓				
7. KOREAN SHOULDER SCORING SYSTEM (KSS) [73]	✓		✓	✓	✓		✓				
8. MELBOURNE INSTABILITY SHOULDER SCALE (MISS) [80]	✓	✓	✓		✓		✓	✓	✓		
9. MODIFIED CONSTANT-MURLEY SCORE [82]	✓		✓		✓		✓	✓	✓		
10. MODIFIED ROWE SHOULDER SCORE (MRS) [88]	✓	✓	✓					✓	✓		
11. MODIFIED UNIVERSITY OF CALIFORNIA—LOS ANGELES SHOULDER SCALE (UCLA) [95]	✓		✓	✓	✓		✓				
12. MUNICH SHOULDER QUESTIONNAIRE (MSQ) [100]	✓		✓		✓		✓	✓	✓	✓	✓
13. OXFORD INSTABILITY SCORE (OIS) [104]		✓	✓		✓		✓	✓	✓	✓	✓
14. OXFORD SHOULDER SCORE (OSS) [111]			✓				✓		✓		
15. PEDIATRIC/ADOLESCENT SHOULDER SURVEY (PASS) [129]	✓	✓	✓		✓	✓		✓		✓	✓
16. PENN SHOULDER SCORE (PSS) [132]	✓		✓	✓			✓	✓	✓		
17. ROTATOR CUFF QUALITY OF LIFE (RC-QOL) [142]	✓		✓		✓		✓	✓	✓	✓	✓
18. ROWE SCALE [94]	✓	✓						✓	✓		
19. SHORT WESTERN ONTARIO ROTATOR CUFF INDEX (SHORTWORC) [159]					✓	✓	✓		✓		
20. SHOULDER ACTIVITY RATING SCALE (SARS) [12]	✓				✓			✓			
21. SHOULDER FUNCTION INDEX (SFInX) [165]					✓		✓	✓			
22. SHOULDER PAIN AND DISABILITY INDEX (SPADI) [167]			✓		✓		✓				
23. SHOULDER PAIN SCORE (SPS) [196]			✓								
24. SHOULDER RATING QUESTIONNAIRE (SRQ) [203]	✓		✓	✓	✓		✓	✓	✓		
25. SIMPLE SHOULDER TEST (SST) [206]	✓				✓		✓	✓	✓		
26. SINGLE ASSESSMENT NUMERIC EVALUATION RATING (SANE) [216]							✓	✓	✓		
27. SUBJECTIVE SHOULDER RATING SCALE (SSRS) [223]	✓	✓	✓					✓	✓		
28. UNIVERSITY OF CALIFORNIA—LOS ANGELES SHOULDER SCALE (UCLA) [225]	✓		✓		✓		✓				
29. UNITED KINGDOM SHOULDER DISABILITY QUESTIONNAIRE (UK-SDQ) [230]			✓				✓	✓			✓
30. WESTERN ONTARIO SHOULDER INSTABILITY INDEX (WOSI) [232]	✓	✓	✓		✓	✓	✓	✓	✓		✓
31. WESTERN ONTARIO OSTEOARTHRITIS OF THE SHOULDER INDEX (WOOS) [250]			✓			✓	✓	✓	✓		✓
32. WESTERN ONTARIO ROTATOR CUFF INDEX (WORC) [258]			✓			✓	✓	✓	✓		✓

Abbreviations: ADL, activities of daily living; ASES, American Shoulder and Elbow Surgeons Standardized Shoulder Assessment Form; CMS, Constant-Murley Score; Dutch-SDQ, Dutch Shoulder Disability Questionnaire; FLEX-SF, Flexilevel Scale of Shoulder Function; FSS, Functional Shoulder Score; MISS, Melbourne Instability Shoulder Scale; MSQ, Munich Shoulder Questionnaire; OIS, Oxford Instability Score; OSS, Oxford Shoulder Score; PSS, Penn Shoulder Score; RC-QOL, Rotator Cuff Quality Of Life; ROM, range of motion; SHORTWORC, Short Western Ontario Rotator Cuff Index; SARS, Shoulder Activity Rating Scale; SFInX, Shoulder Function Index; SPADI, Shoulder Pain and Disability Index; SPS, Shoulder Pain Score; SRQ, Shoulder Rating Questionnaire; SST, Simple Shoulder Test; SANE, Single Assessment Numeric Evaluation Rating; SRSS, Subjective Shoulder Rating Scale; UCLA, University of California—Los Angeles Shoulder Scale; UK-SDQ, United Kingdom Shoulder Disability Questionnaire; WOSI, Western Ontario Shoulder Instability Index; WOOS, Western Ontario Osteoarthritis of the Shoulder index; WORC, Western Ontario Rotator Cuff Index.

## Abbreviations

ADL, activities of daily living; ASES, American Shoulder and Elbow Surgeons Standardized Shoulder Assessment Form; CMS, Constant-Murley Score; Dutch-SDQ, Dutch Shoulder Disability Questionnaire; FLEX-SF, Flexilevel Scale of Shoulder Function; FSS, Functional Shoulder Score; MISS, Melbourne Instability Shoulder Scale; MSQ, Munich Shoulder Questionnaire; OIS, Oxford Instability Score; OSS, Oxford Shoulder Score; PROMs, patient reported outcome measures; PSS, Penn Shoulder Score; RC-QOL, Rotator Cuff Quality Of Life; ROM, range of motion; SHORTWORC, Short Western Ontario Rotator Cuff Index; SARS, Shoulder Activity Rating Scale; SFInX, Shoulder Function Index; SPADI, Shoulder Pain and Disability Index; SPS, Shoulder Pain Score; SRQ, Shoulder Rating Questionnaire; SST, Simple Shoulder Test; SANE, Single Assessment Numeric Evaluation Rating; SRSS, Subjective Shoulder Rating Scale; UCLA, University of California—Los Angeles Shoulder Scale; UK-SDQ, United Kingdom Shoulder Disability Questionnaire; WOSI, Western Ontario Shoulder Instability Index; WOOS, Western Ontario Osteoarthritis of the Shoulder index; WORC, Western Ontario Rotator Cuff Index.

## References

[1] Goetti P., Denard P.J., Collin P., Ibrahim M., Hoffmeyer P., Lädermann A. (2020). Shoulder Biomechanics in Normal and Selected Pathological Conditions. EFORT Open Rev..

[2] Urwin M., Symmons D., Allison T., Brammah T., Busby H., Roxby M., Simmons A., Williams G. (1998). Estimating the Burden of Musculoskeletal Disorders in the Community: The Comparative Prevalence of Symptoms at Different Anatomical Sites, and the Relation to Social Deprivation. Ann. Rheum. Dis..

[3] Buck F.M., Jost B., Hodler J. (2008). Shoulder Arthroplasty. Eur. Radiol..

[4] Thigpen C.A., Shaffer M.A., Gaunt B.W., Leggin B.G., Williams G.R., Wilcox R.B. (2016). The American Society of Shoulder and Elbow Therapists’ Consensus Statement on Rehabilitation Following Arthroscopic Rotator Cuff Repair. J. Shoulder Elb. Surg..

[5] Best M.J., Tanaka M.J. (2018). Multidirectional Instability of the Shoulder: Treatment Options and Considerations. Sports Med. Arthrosc..

[6] Ropars M., Thomazeau H., Huten D. (2017). Clavicle Fractures. Orthop. Traumatol. Surg. Res..

[7] Launonen A.P., Lepola V., Saranko A., Flinkkilä T., Laitinen M., Mattila V.M. (2015). Epidemiology of Proximal Humerus Fractures. Arch. Osteoporos..

[8] Edwards P., Ebert J., Joss B., Bhabra G., Ackland T., Wang A. (2016). Exercise Rehabilitation in the Non-Operative Management of Rotator Cuff Tears: A Review of the Literature. Int. J. Sports Phys. Ther..

[9] Innocenti T., Ristori D., Miele S., Testa M. (2019). The Management of Shoulder Impingement and Related Disorders: A Systematic Review on Diagnostic Accuracy of Physical Tests and Manual Therapy Efficacy. J. Bodyw. Mov. Ther..

[10] Vicente-Herrero M.T., Capdevila García L., López González Á.A., Ramírez Iñiguez de la Torre M.V. (2009). El Hombro y Sus Patologías En Medicina Del Trabajo. Semer. Med. Fam..

[11] Michener L.A., Leggin B.G. (2001). A Review of Self-Report Scales for the Assessment of Functional Limitation and Disability of the Shoulder. J. Hand Ther..

[12] Brophy R.H., Beauvais R.L., Jones E.C., Cordasco F.A., Marx R.G. (2005). Measurement of Shoulder Activity Level. Clin. Orthop. Relat. Res..

[13] Chamorro-Moriana G., Ridao-Fernández C., Ojeda J., Benítez-Lugo M., Sevillano J.L. (2016). Reliability and Validity Study of the Chamorro Assisted Gait Scale for People with Sprained Ankles, Walking with Forearm Crutches. PLoS ONE.

[14] Ware J.E., Brook R.H., Davies A.R., Lohr K.N. (1981). Choosing Measures of Health Status for Individuals in General Populations. Am. J. Public Health.

[15] Moher D., Liberati A., Tetzlaff J., Altman D.G., Grp P. (2009). Preferred Reporting Items for Systematic Reviews and Meta-Analyses: The PRISMA Statement. PLoS Med..

[16] Whiting P.F., Rutjes A.W., Westwood M.E., Mallett S., Deeks J.J., Reitsma J.B., Leeflang M.M., Sterne J.A., Bossuyt P.M., QUADAS-2, Grp (2011). QUADAS-2: A Revised Tool for the Quality Assessment of Diagnostic Accuracy Studies. Ann. Intern. Med..

[17] Mokkink L.B., de Vet H.C.W., Prinsen C.A.C., Patrick D.L., Alonso J., Bouter L.M., Terwee C.B. (2018). COSMIN Risk of Bias Checklist for Systematic Reviews of Patient-Reported Outcome Measures. Qual. Life Res..

[18] Møller A., Bissenbakker K.H., Arreskov A.B., Brodersen J. (2020). Specific Measures of Quality of Life in Patients with Multimorbidity in Primary Healthcare: A Systematic Review on Patient-Reported Outcome Measures’ Adequacy of Measurement. Patient Relat. Outcome Meas..

[19] Richards R.R., An K.N., Bigliani L.U., Friedman R.J., Gartsman G.M., Gristina A.G., Iannotti J.P., Mow V.C., Sidles J.A., Zuckerman J.D. (1994). A Standardized Method for the Assessment of Shoulder Function. J. Shoulder Elb. Surg..

[20] Beaton D.E., Richards R.R. (1996). Measuring Function of the Shoulder. A Cross-Sectional Comparison of Five Questionnaires. J. Bone Jt. Surg. Am..

[21] Beaton D., Richards R.R. (1998). Assessing the Reliability and Responsiveness of 5 Shoulder Questionnaires. J. Shoulder Elb. Surg..

[22] Cook K.F., Roddey T.S., Olson S.L., Gartsman G.M., Valenzuela F.F.T., Hanten W.P. (2002). Reliability by Surgical Status of Self-Reported Outcomes in Patients Who Have Shoulder Pathologies. J. Orthop. Sports Phys. Ther..

[23] Michener L.A., McClure P.W., Sennett B.J. (2002). American Shoulder and Elbow Surgeons Standardized Shoulder Assessment Form, Patient Self-Report Section: Reliability, Validity, and Responsiveness. J. Shoulder Elb. Surg..

[24] Oh J.H., Jo K.H., Kim W.S., Gong H.S., Han S.G., Kim Y.H. (2009). Comparative Evaluation of the Measurement Properties of Various Shoulder Outcome Instruments. Am. J. Sports Med..

[25] Kemp K.A.R., Sheps D.M., Beaupre L.A., Styles-Tripp F., Luciak-Corea C., Balyk R. (2012). An Evaluation of the Responsiveness and Discriminant Validity of Shoulder Questionnaires among Patients Receiving Surgical Correction of Shoulder Instability. Sci. World J..

[26] Sciascia A.D., Morris B.J., Jacobs C.A., Edwards T.B. (2017). Responsiveness and Internal Validity of Common Patient-Reported Outcome Measures Following Total Shoulder Arthroplasty. Orthopedics.

[27] Dabija D.I., Pennings J.S., Archer K.R., Ayers G.D., Higgins L.D., Kuhn J.E., Baumgarten K.M., Matzkin E., Jain N.B. (2019). Which Is the Best Outcome Measure for Rotator Cuff Tears?. Clin. Orthop. Relat. Res..

[28] Vrotsou K., Cuéllar R., Silió F., Garay D., Busto G., Escobar A. (2019). Test-Retest Reliability of the ASES-p Shoulder Scale. Musculoskelet. Sci. Pract..

[29] Gotlin M.J., Kingery M.T., Baron S.L., McCafferty J., Jazrawi L.M., Meislin R.J. (2020). Recall Bias in Retrospective Assessment of Preoperative Patient-Reported American Shoulder and Elbow Surgeons Scores in Arthroscopic Rotator Cuff Repair Surgery. Am. J. Sports Med..

[30] Hou J., Li Q., Yu M., Li F., Tang Y., Long Y., Alike Y., Zhang Y., Ali M.I., Zhang C. (2020). Validation of a Mobile Version of the American Shoulder and Elbow Surgeons Standardized Shoulder Assessment Form: An Observational Randomized Crossover Trial. JMIR mHealth uHealth.

[31] Baumgarten K.M., Chang P.S. (2020). The American Shoulder and Elbow Surgeons Score Highly Correlates with the Simple Shoulder Test. J. Shoulder Elb. Surg..

[32] Zabrzyński J., Paczesny Ł., Łapaj Ł., Zabrzyńska A., Szwedowski D. (2020). The Surgical Treatment of the Long Head of Biceps Tendon and the Autotenodesis Phenomenon: An Ultrasound and Arthroscopic Study. Folia Morphol..

[33] Howard L., Berdusco R., Momoli F., Pollock J., Liew A., Papp S., Lalonde K.-A., Gofton W., Ruggiero S., Lapner P. (2018). Open Reduction Internal Fixation vs Non-Operative Management in Proximal Humerus Fractures: A Prospective, Randomized Controlled Trial Protocol. BMC Musculoskelet. Disord..

[34] Provencher M.T., Kirby H., McDonald L.S., Golijanin P., Gross D., Campbell K.J., LeClere L., Sanchez G., Anthony S., Romeo A.A. (2017). Surgical Release of the Pectoralis Minor Tendon for Scapular Dyskinesia and Shoulder Pain. Am. J. Sports Med..

[35] Goldhahn J., Angst F., Drerup S., Pap G., Simmen B.R., Mannion A.F. (2008). Lessons Learned during the Cross-Cultural Adaptation of the American Shoulder and Elbow Surgeons Shoulder Form into German. J. Shoulder Elb. Surg..

[36] Padua R., Padua L., Ceccarelli E., Bondi R., Alviti F., Castagna A. (2010). Italian Version of ASES Questionnaire for Shoulder Assessment: Cross-Cultural Adaptation and Validation. Musculoskelet. Surg..

[37] Yahia A., Guermazi M., Khmekhem M., Ghroubi S., Ayedi K., Elleuch M.H. (2011). Traduction En Arabe et Validation de l’indice ASES Dans l’évaluation de l’incapacité Fonctionnelle Des Pathologies de l’épaule. Ann. Phys. Rehabil. Med..

[38] Celik D., Atalar A.C., Demirhan M., Dirican A. (2013). Translation, cultural adaptation, validity and reliability of the Turkish ASES questionnaire. Knee Surg. Sports Traumatol. Arthrosc..

[39] Felsch Q.T.M., Sievert P., Schotanus M.G.M., Jansen E.J.P. (2019). The Dutch Version of the American Shoulder and Elbow Surgeons Standardized Shoulder Assessment Form Is a Reliable and Valid Questionnaire for Shoulder Problems. JSES Open Access.

[40] Piitulainen K., Paloneva J., Ylinen J., Kautiainen H., Häkkinen A. (2014). Reliability and Validity of the Finnish Version of the American Shoulder and Elbow Surgeons Standardized Shoulder Assessment Form, Patient Self-Report Section. BMC Musculoskelet. Disord..

[41] De Lima Moser A.D., Knaut L.A.M., Zotz T.G., Scharan K.O. (2012). Validade e Confiabilidade Da Versão Em Português Do American Shoulder and Elbow Surgeons Standardized Shoulder Assessment Form. Rev. Bras. Reumatol..

[42] Policastro P.O., Pierobon A., Pérez J., Novoa G.A., Calvo Delfino M., Sajfar M.E., Salzberg S., Carmody C., Dorado J.H., Raguzzi I. (2019). Cross-Cultural Adaptation and Validation of the Argentine “American Shoulder and Elbow Surgeons, Patient Self-Report Section” Questionnaire. Musculoskelet. Sci. Pract..

[43] Vrotsou K., Cuéllar R., Silió F., Rodriguez M.Á., Garay D., Busto G., Trancho Z., Escobar A. (2016). Patient Self-Report Section of the ASES Questionnaire: A Spanish Validation Study Using Classical Test Theory and the Rasch Model. Heal. Qual. Life Outcomes.

[44] Constant C.R., Murley A.H. (1987). A Clinical Method of Functional Assessment of the Shoulder. Clin. Orthop. Relat. Res..

[45] Conboy V.B., Morris R.W., Kiss J., Carr A.J. (1996). An Evaluation of the Constant-Murley Shoulder Assessment. J. Bone Jt. Surg. Br..

[46] Angst F., Goldhahn J., Drerup S., Aeschlimann A., Schwyzer H.K., Simmen B.R. (2008). Responsiveness of Six Outcome Assessment Instruments in Total Shoulder Arthroplasty. Arthritis Care Res..

[47] Razmjou H., Bean A., MacDermid J.C., van Osnabrugge V., Travers N., Holtby R. (2008). Convergent Validity of the Constant-Murley Outcome Measure in Patients with Rotator Cuff Disease. Physiother. Canada.

[48] Rocourt M.H.H., Radlinger L., Kalberer F., Sanavi S., Schmid N.S., Leunig M., Hertel R. (2008). Evaluation of Intratester and Intertester Reliability of the Constant-Murley Shoulder Assessment. J. Shoulder Elb. Surg..

[49] Ban I., Troelsen A., Kristensen M.T. (2016). High Inter-Rater Reliability, Agreement, and Convergent Validity of Constant Score in Patients with Clavicle Fractures. J. Shoulder Elb. Surg..

[50] Mahabier K.C., Den Hartog D., Theyskens N., Verhofstad M.H.J., Van Lieshout E.M.M., HUMMER Trial Investigators (2017). Reliability, Validity, Responsiveness, and Minimal Important Change of the Disablities of the Arm, Shoulder and Hand and Constant-Murley Scores in Patients with a Humeral Shaft Fracture. J. Shoulder Elb. Surg..

[51] James-Belin E., Roy A.L., Lasbleiz S., Ostertag A., Yelnik A., Orcel P., Beaudreuil J. (2019). Comparative Study of Psychometric Properties of Three Assessment Tools for Degenerative Rotator Cuff Disease. Clin. Rehabil..

[52] Hallgren H.C.B., Adolfsson L.E., Johansson K., Oberg B., Peterson A., Holmgren T.M. (2017). Specific Exercises for Subacromial Pain Good Results Maintained for 5 Years. Acta Orthop..

[53] Yao M., Yang L., Cao Z.-Y., Cheng S.D., Tian S.L., Sun Y.-L., Wang J., Xu B.-P., Hu X.C., Wang Y.J. (2017). Chinese Version of the Constant-Murley Questionnaire for Shoulder Pain and Disability: A Reliability and Validation Study. Health Qual. Life Outcomes.

[54] Livain T., Pichon H., Vermeulen J., Vaillant J., Saragaglia D., Poisson M.F., Monnet S. (2007). Étude de Reproductibilité Intra et Inter-Observateur de La Version Française Du Score de Constant Au Cours de La Rééducation Des Coiffes Opérées. Rev. Chir. Orthop. Reparatrice Appar. Mot..

[55] Barreto R.P.G., Barbosa M.L.L., Balbinotti M.A.A., Mothes F.C., da Rosa L.H.T., Silva M.F. (2016). The Brazilian Version of the Constant-Murley Score (CMS-BR): Convergent and Construct Validity, Internal Consistency, and Unidimensionality. Rev. Bras. Ortop..

[56] Carosi M., Galeoto G., Gennaro S.D., Berardi A., Valente D., Servadio A. (2020). Transcultural Reliability and Validity of an Italian Language Version of the Constant–Murley Score. J. Orthop. Trauma Rehabil..

[57] Maqdes A., Hanna S.S., Bouhamra A.K., Khaja A.F. (2020). Cross-Cultural Adaptation and Translation of the Constant Murley Score into Arabic. Sicot-J.

[58] Van der Heijden G.J.M.G. (1996). Shoulder Disability Questionnaire: Design and Responsiveness of a Functional Status Measure. Shoulder Disorder Treatment: Efficacy of Ultrasound Electrotherapy.

[59] Van Der Windt D.A., van Der Heijden G.J., de Winter A.F., Koes B.W., Devillé W., Bouter L.M. (1998). The Responsiveness of the Shoulder Disability Questionnaire. Ann. Rheum. Dis..

[60] Van Der Heijden G.J., Leffers P., Bouter L.M. (2000). Shoulder Disability Questionnaire Design and Responsiveness of a Functional Status Measure. J. Clin. Epidemiol..

[61] Paul A., Lewis M., Shadforth M.F., Croft P.R., Van Der Windt D.A., Hay E.M. (2004). A Comparison of Four Shoulder-Specific Questionnaires in Primary Care. Ann. Rheum. Dis..

[62] Alvarez-Nemegyei J., Puerto-Ceballos I., Guzmán-Hau W., Bassol-Perea A., Nuño-Gutiérrez B.L. (2005). Development of a Spanish-Language Version of the Shoulder Disability Questionnaire. J. Clin. Rheumatol..

[63] Cook K.F., Roddey T.S., Gartsman G.M., Olson S.L. (2003). Development and Psychometric Evaluation of the Flexilevel Scale of Shoulder Function. Med. Care.

[64] Lin J.J., Lim H.K., Soto-quijano D.A., Hanten W.P., Olson S.L., Roddey T.S., Sherwood A.M. (2006). Altered Patterns of Muscle Activation during Performance of Four Functional Tasks in Patients with Shoulder Disorders: Interpretation from Voluntary Response Index. J. Electromyogr. Kinesiol..

[65] Yang J.L., Chang C.W., Chen S.Y., Lin J.J. (2008). Shoulder Kinematic Features Using Arm Elevation and Rotation Tests for Classifying Patients with Frozen Shoulder Syndrome Who Respond to Physical Therapy. Man. Ther..

[66] Yang J.L., Lu T.W., Chou F.C., Chang C.W., Lin J.J. (2009). Secondary Motions of the Shoulder during Arm Elevation in Patients with Shoulder Tightness. J. Electromyogr. Kinesiol..

[67] Agarwal S., Raza S., Moiz J., Anwer S., Alghadir A.H. (2016). Effects of Two Different Mobilization Techniques on Pain, Range of Motion and Functional Disability in Patients with Adhesive Capsulitis: A Comparative Study. J. Phys. Ther. Sci..

[68] Hung C.J., Jan M.H., Lin Y.F., Wang T.Q., Lin J.J. (2010). Scapular Kinematics and Impairment Features for Classifying Patients with Subacromial Impingement Syndrome. Man. Ther..

[69] Agarwal S. (2012). Healing Rates for Challenging Rotator Cuff Tears Utilizing an Acellular Human Dermal Reinforcement Graft. Int. J. Shoulder Surg..

[70] Ge Y., Chen S., Chen J., Hua Y., Li Y. (2013). The Development and Evaluation of a New Shoulder Scoring System Based on the View of Patients and Physicians: The Fudan University Shoulder Score. Arthrosc. J. Arthrosc. Relat. Surg..

[71] Li H., Chen Y., Chen S. (2019). Postoperative Residual Pain Is Associated with a High Magnetic Resonance Imaging (MRI)-Based Signal Intensity of the Repaired Supraspinatus Tendon. Knee Surg. Sport. Traumatol. Arthrosc..

[72] Iossifidis A., Ibrahim E.F., Petrou C., Galanos A. (2015). The Development and Validation of a Questionnaire for Rotator Cuff Disorders: The Functional Shoulder Score. Shoulder Elbow.

[73] Tae S.K., Rhee Y.G., Park T.S., Lee K.W., Park J.Y., Choi C.H., Koh S.H., Oh J.H., Kim S.Y., Shin S.J. (2009). The Development and Validation of an Appraisal Method for Rotator Cuff Disorders: The Korean Shoulder Scoring System. J. Shoulder Elb. Surg..

[74] Kim H., Park H.J., Lee S.Y., Kim J.N., Moon J., Kim M.S., Kim E. (2020). Ultrasound Evaluation of Postsurgical Shoulder after Rotator Cuff Repair: Comparison of Clinical Results. Acta Radiol..

[75] Kim I.-B., Jung D.W. (2018). An Intra-Articular Steroid Injection at 6 Weeks Postoperatively for Shoulder Stiffness After Arthroscopic Rotator Cuff Repair Does Not Affect Repair Integrity. Am. J. Sports Med..

[76] Kim I.-B., Jung D.-W. (2018). A Rotator Cuff Tear Concomitant With Shoulder Stiffness Is Associated With a Lower Retear Rate After 1-Stage Arthroscopic Surgery. Am. J. Sports Med..

[77] Cho N.S., Bae S.J., Lee J.W., Seo J.H., Rhee Y.G. (2019). Clinical and Radiological Outcomes of Modified Phemister Operation with Coracoclavicular Ligament Augmentation Using Suture Anchor for Acute Acromioclavicular Joint Dislocation. Clin. Shoulder Elb..

[78] Choi C.-H., Jun C.-M., Kim J.-Y. (2019). A Comparative Study on Internal Fixation Using Long Proximal Intramedullary Nail for the Treatment of Humeral Shaft Fracture According to Fracture Types. Clin. Shoulder Elb..

[79] Jung T.W., Lee S.Y., Min S.K., Lee S.M., Yoo J.C. (2019). Does Combining a Suprascapular Nerve Block With an Intra-Articular Corticosteroid Injection Have an Additive Effect in the Treatment of Adhesive Capsulitis? A Comparison of Functional Outcomes After Short-Term and Minimum 1-Year Follow-Up. Orthop. J. Sport. Med..

[80] Watson L., Story I., Dalziel R., Hoy G., Shimmin A., Woods D. (2005). A New Clinical Outcome Measure of Glenohumeral Joint Instability: The MISS Questionnaire. J. Shoulder Elb. Surg..

[81] Taylor D., Garewal D., Evans M.C. (2015). Correlations between Three Patient-Assessed Shoulder Instability Scales. J. Orthop. Surg..

[82] Constant C.R., Gerber C., Emery R.J.H., Søjbjerg J.O., Gohlke F., Boileau P. (2008). A Review of the Constant Score: Modifications and Guidelines for Its Use. J. Shoulder Elb. Surg..

[83] Van De Water A.T.M., Shields N., Davidson M., Evans M., Taylor N.F. (2014). Reliability and Validity of Shoulder Function Outcome Measures in People with a Proximal Humeral Fracture. Disabil. Rehabil..

[84] Moeller A.D., Thorsen R.R., Torabi T.P., Bjoerkman A.S., Christensen E.H., Maribo T., Christiansen D.H. (2014). The Danish Version of the Modified Constant-Murley Shoulder Score: Reliability, Agreement, and Construct Validity. J. Orthop. Sports Phys. Ther..

[85] Ntourantonis D., Panagopoulos A., Iliopoulos I., Tatani I., Tsoumpos P., Kouzelis A., Tyllianakis M. (2017). Translation, Cultural Adaptation, Validity and Reliability of the Greek Version of the Modified Constant Score. JSES Open Access.

[86] Coory J.A., Parr A.F., Wilkinson M.P., Gupta A. (2019). Efficacy of Suprascapular Nerve Block Compared with Subacromial Injection: A Randomized Controlled Trial in Patients with Rotator Cuff Tears. J. Shoulder Elb. Surg..

[87] Çelik D. (2016). Turkish Version of the Modified Constant-Murley Score and Standardized Test Protocol: Reliability and Validity. Acta Orthop. Traumatol. Turc..

[88] Rowe C.R., Zarins B. (1981). Recurrent Transient Subluxation of the Shoulder. J. Bone Jt. Surg. Ser. A.

[89] Romeo A.A., Bach B.R., O’Halloran K.L. (1996). Scoring Systems for Shoulder Conditions. Am. J. Sports Med..

[90] Rubenstein D.L., Jobe F.W., Glousman R.E., Kvitne R.S., Pink M., Giangarra C.E. (1992). Anterior Capsulolabral Reconstruction of the Shoulder in Athletes. J. Shoulder Elb. Surg..

[91] Fjalestad T., Strømsøe K., Blücher J., Tennøe B. (2005). Fractures in the Proximal Humerus: Functional Outcome and Evaluation of 70 Patients Treated in Hospital. Arch. Orthop. Trauma Surg..

[92] El Shewy M.T., El Barbary H.M., El Meligy Y.H., Khaled S.A. (2008). Open Reduction and Posterior Capsular Shift for Cases of Neglected Unreduced Posterior Shoulder Dislocation. Am. J. Sports Med..

[93] Marcondes F.B., de Vasconcelos R.A., Marchetto A., de Andrade A.L.L., Filho A.Z., Etchebehere M. (2012). Translation To Portuguese Language and Cross-Cultural Adaptation of the Modified Rowe Score for Overhead Athletes. Rev. Bras. Ortop..

[94] Rowe C.R., Patel D., Southmayd W.W. (1978). The Bankart Procedure: A Long-Term End Result Study. J. Bone Jt. Surg..

[95] Ellman H., Hanker G., Bayer M. (1986). Repair of the Rotator Cuff. End-Result Study of Factors Influencing Reconstruction. J. Bone Jt. Surg. Am..

[96] Vascellari A., Venturin D., Ramponi C., Ben G., Poser A., Rossi A., Coletti N. (2018). Psychometric Properties of Three Different Scales for Subjective Evaluation of Shoulder Pain and Dysfunction in Italian Patients after Shoulder Surgery for Anterior Instability. J. Shoulder Elb. Surg..

[97] Malavolta E.A., Assunção J.H., Gracitelli M.E.C., Simões P.A.A., Shido D.K., Ferreira Neto A.A. (2018). Correlation between the UCLA and Constant-Murley Scores in Rotator Cuff Repairs and Proximal Humeral Fractures Osteosynthesis. Rev. Bras. Ortop..

[98] Lim K.K., Chang H.C., Tan J.L., Chan B.K. (2007). Arthroscopic Subacromial Decompression for Stage-II Impingement. J. Orthop. Surg..

[99] Marchese C., Cristalli G., Pichi B., Manciocco V., Mercante G., Pellini R., Marchesi P., Sperduti I., Ruscito P., Spriano G. (2012). Italian Cross-Cultural Adaptation and Validation of Three Different Scales for the Evaluation of Shoulder Pain and Dysfunction after Neck Dissection. Acta Otorhinolaryngol. Ital..

[100] Schmidutz F., Beirer M., Braunstein V., Bogner V., Wiedemann E., Biberthaler P. (2012). The Munich Shoulder Questionnaire (MSQ): Development and Validation of an Effective Patient-Reported Tool for Outcome Measurement and Patient Safety in Shoulder Surgery. Patient Saf. Surg..

[101] Greve F., Beirer M., Zyskowski M., Crönlein M., Müller M., Pesch S., Felix S., Biberthaler P., Buchholz A., Kirchhoff C. (2019). Prospective Outcome Analysis Following Tenodesis of the Long Head of the Biceps Tendon along with Locking Plate Osteosynthesis for Proximal Humerus Fractures. Injury.

[102] Biberthaler P., Beirer M., Kirchhoff S. (2013). Significant Benefit for Older Patients after Arthroscopic Subacromial Decompression: A Long-Term Follow-up Study. Int Orthop..

[103] Beirer M., Siebenlist S., Crönlein M., Postl L., Huber-Wagner S., Biberthaler P. (2014). Clinical and Radiological Outcome Following Treatment of Displaced Lateral Clavicle Fractures Using a Locking Compression Plate with Lateral Extension: A Prospective Study. BMC Musculoskelet. Disord..

[104] Dawson J., Fitzpatrick R., Carr A. (1999). The Assessment of Shoulder Instability. The Development and Validation of a Questionnaire. J. Bone J. Surg. Br..

[105] Van der Linde J.A., van Kampen D.A., van Beers L.W.A.H., van Deurzen D.F.P., Saris D.B.F., Terwee C.B. (2017). The Responsiveness and Minimal Important Change of the Western Ontario Shoulder Instability Index and Oxford Shoulder Instability Score. J. Orthop. Sport. Phys. Ther..

[106] Van der Linde J.A., van Kampen D.A., van Beers L.W.A.H., van Deurzen D.F.P., Terwee C.B., Willems W.J. (2015). The Oxford Shoulder Instability Score; Validation in Dutch and First-Time Assessment of Its Smallest Detectable Change. J. Orthop. Surg. Res..

[107] Flinkkilä T., Hyvönen P., Ohtonen P., Leppilahti J. (2010). Arthroscopic Bankart Repair: Results and Risk Factors of Recurrence of Instability. Knee Surg. Sport. Traumatol. Arthrosc..

[108] Schrøder C.P., Skare Ø., Reikerås O., Mowinckel P., Brox J.I. (2017). Sham Surgery versus Labral Repair or Biceps Tenodesis for Type II SLAP Lesions of the Shoulder: A Three-Armed Randomised Clinical Trial. Br. J. Sports Med..

[109] Özden F., Mazzoni B., Cucchi D., Giovannelli T., Paci M., Arrigoni P., Nicoletti S., Ozden F. (2019). Translation, Cross-Cultural Adaptation, and Validation of the Italian Version of the Oxford Shoulder Instability Score. Int. Orthop..

[110] Sonmezer E., Yosmaoglu H.B., Doğan C.D. (2020). The Reliability and Validity of the Turkish Version of the Oxford Shoulder Instability Score. Disabil. Rehabil..

[111] Dawson J., Fitzpatrick R., Carr A. (1996). Questionnaire on the Perceptions of Patients about Shoulder Surgery. J. Bone Jt. Surg. Ser. B.

[112] Huber W., Hofstaetter J.G., Hanslik-Schnabel B., Posch M., Wurnig C. (2004). The German Version of the Oxford Shoulder Score Cross-Cultural Adaptation and Validation. Arch. Orthop. Trauma Surg..

[113] Haragus H., Prejbeanu R., Patrascu J., Faur C., Roman M., Melinte R., Timar B., Codorean I., Stetson W., Marra G. (2018). Cross-Cultural Adaptation and Validation of the Romanian Oxford Shoulder Score. Medicine.

[114] Tuton D., Barbe C., Salmon J.-H., Drame M., Nerot C., Ohl X. (2016). Transcultural Validation of the Oxford Shoulder Score for the French-Speaking Population. Orthop. Traumatol. Res..

[115] Da Lima E.S., Natour J., Moreira E., Jones A. (2016). Translation, Cultural Adaptation and Reproducibility of the Oxford Shoulder Score Questionnaire for Brazil, among Patients with Rheumatoid Arthritis. Sao Paulo Med. J..

[116] Kraal T., van der Meer O., van den Borne M., Koenraadt K., Eygendaal D., Boer R. (2019). Manipulation under Anesthesia for Frozen Shoulders: A Retrospective Cohort Study. Acta Orthop. Belg..

[117] Goncalves R.S., Caldeira C.Q., Rodrigues V.M., Felicia S.C., Cavalheiro L.M., Ferreira P.L. (2018). Cross-Cultural Adaptation and Validation of the Portuguese Version of the Oxford Shoulder Score (OSS). Acta Reumatol. Port..

[118] Bejer A., Szczepanik M., Płocki J., Szymczyk D., Kulczyk M., Pop T. (2019). Translation, Cross-Cultural Adaptation and Validation of the Polish Version of the Oxford Shoulder Score in Patients Undergoing Arthroscopic Rotator Cuff Repair. Health Qual. Life Outcomes.

[119] Tuǧay U., Tuǧay N., Gelecek N., Özkan M. (2011). Oxford Shoulder Score: Cross-Cultural Adaptation and Validation of the Turkish Version. Arch. Orthop. Trauma Surg..

[120] Roh Y.H., Noh J.H., Kim W., Oh J.H., Gong H.S., Baek G.H. (2012). Cross-Cultural Adaptation and Validation of the Korean Version of the Oxford Shoulder Score. Arch. Orthop. Trauma Surg..

[121] Xu X., Wang F., Wang X., Wei X., Wang Z. (2015). Chinese Cross-Cultural Adaptation and Validation of the Oxford Shoulder Score. Health Qual. Life Outcomes.

[122] Murena L., Vulcano E., D’Angelo F., Monti M., Cherubino P. (2010). Italian Cross-Cultural Adaptation and Validation of the Oxford Shoulder Score. J. Shoulder Elb. Surg..

[123] Berendes T., Pilot P., Willems J., Verburg H., te Slaa R. (2010). Validation of the Dutch Version of the Oxford Shoulder Score. J. Shoulder Elb. Surg..

[124] Ebrahimzadeh M.H., Birjandinejad A., Razi S., Mardani-Kivi M., Kachooei A.R. (2015). Oxford Shoulder Score: A Cross-Cultural Adaptation and Validation Study of the Persian Version in Iran. Iran. J. Med. Sci..

[125] Frich L.H., Noergaard P.M., Brorson S. (2011). Validation of the Danish Version of Oxford Shoulder Score. Dan. Med. Bull..

[126] Ekeberg O.M., Bautz-Holter E., Tveitå E.K., Keller A., Juel N.G., Brox J.I. (2008). Agreement, Reliability and Validity in 3 Shoulder Questionnaires in Patients with Rotator Cuff Disease. BMC Musculoskelet. Disord..

[127] Alsanawi H.A., Alghadir A.H., Anwer S., Alenazi H.A., Li H. (2020). Internal Consistency, Test–Retest Reliability, and Construct Validity of the Adapted Arabic Version of the Oxford Shoulder Score in Patients with Shoulder Disorders. Disabil. Rehabil..

[128] Torres-Lacomba M., Sánchez-Sánchez B., Prieto-Gómez V., Pacheco-da-Costa S., Yuste-Sánchez M.J., Navarro-Brazález B., Gutiérrez-Ortega C. (2015). Spanish Cultural Adaptation and Validation of the Shoulder Pain and Disability Index, and the Oxford Shoulder Score after Breast Cancer Surgery. Health Qual. Life Outcomes.

[129] Edmonds E.W., Bastrom T.P., Roocroft J.H., Calandra-Young V.A., Pennock A.T. (2017). The Pediatric/Adolescent Shoulder Survey (PASS) A Reliable Youth Questionnaire with Discriminant Validity and Responsiveness to Change. Orthop. J. Sport. Med..

[130] Hughes J.L., Bastrom T., Pennock A.T., Edmonds E.W. (2018). Arthroscopic Bankart Repairs With and Without Remplissage in Recurrent Adolescent Anterior Shoulder Instability with Hill-Sachs Deformity. Orthop. J. Sport. Med..

[131] Hansen C.H., Asturias A.M., Pennock A.T., Edmonds E.W. (2020). Adolescent Posterior-Superior Glenoid Labral Pathology: Does Involvement of the Biceps Anchor Make a Difference?. Am. J. Sports Med..

[132] Leggin B.G., Iannoti J.P., Williams G.R. (1999). Shoulder Outcome Measurement. Disorders of the Shoulder: Diagnosis and Management.

[133] Cook K.F., Gartsman G.M., Roddey T.S., Olson S.L. (2001). The Measurement Level and Trait-Specific Reliability of 4 Scales of Shoulder Functioning: An Empiric Investigation. Arch. Phys. Med. Rehabil..

[134] Leggin B.G., Michener L.A., Shaffer M.A., Brenneman S.K., Iannotti J.P., Williams G.R. (2006). The Penn Shoulder Score: Reliability and Validity. J. Orthop. Sports Phys. Ther..

[135] Hazar Kanik Z., Gunaydin G., Pala O.O., Sozlu U., Alkan Z.B., Citaker S., Basar S., Kanatli U. (2018). Translation, Cultural Adaptation, Reliability, and Validity of the Turkish Version of the Penn Shoulder Score. Disabil. Rehabil..

[136] Coviello J.P., Kakar R.S., Reynolds T.J. (2017). Short-term effects of instrument-assisted soft tissue mobilization on pain free range of motion in a weightlifter with subacromial pain syndrome. Int. J. Sports Phys. Ther..

[137] Roberson T.A., Shanley E., Abildgaard J.T., Granade C.M., Adams K.J., Griscom J.T., Hunt Q., Nix Q., Kissenberth M.J., Tolan S.J. (2019). The Influence of Radiographic Markers of Biomechanical Variables on Outcomes in Reverse Shoulder Arthroplasty. JSES Open Access.

[138] Nicholas S.J., Lee S.J., Mullaney M.J., Tyler T.F., Fukunaga T., Johnson C.D., McHugh M.P. (2016). Functional Outcomes After Double-Row Versus Single-Row Rotator Cuff Repair: A Prospective Randomized Trial. Orthop. J. Sport. Med..

[139] Tate A.R., McClure P., Kareha S., Irwin D., Barbe M.F. (2009). A Clinical Method for Identifying Scapular Dyskinesis, Part 2: Validity. J. Athl. Train..

[140] Napoles B.V., Hoffman C.B., Martins J., de Oliveira A.S. (2010). Translation and Cultural Adaptation of the Penn Shoulder Score to Portuguese Language: PSS-Brazil. Rev. Bras. Reumatol..

[141] De Souza M.B., Martins J., Hotta G.H., De Oliveira A.S. (2015). Measurement Properties of the Brazilian Version of the Penn Shoulder Score (PSS-Brazil): Reliability, Validity, and Responsiveness. J. Orthop. Sports Phys. Ther..

[142] Hollinshead R.M., Mohtadi G.H. (2000). Two 6-Year Follow-up Studies of Large and Massive Rotator Cuff Tears: Comparison of Outcome Measures. J. Shoulder Elb. Surg..

[143] Razmjou H., Bean A., van Osnabrugge V., MacDermid J.C., Holtby R. (2006). Cross-Sectional and Longitudinal Construct Validity of Two Rotator Cuff Disease-Specific Outcome Measures. BMC Musculoskelet. Disord..

[144] Eubank B.H., Mohtadi N.G., Lafave M.R., Wiley J.P., Emery J.C.H. (2017). Further Validation and Reliability Testing of the Rotator Cuff Quality of Life Index (RC-QOL) According to the Consensus-Based Standards for the Selection of Health Measurement Instruments (COSMIN) Guidelines. J. Shoulder Elb. Surg..

[145] Boorman R.S., More K.D., Hollinshead R.M., Wiley J.P., Mohtadi N.G., Lo I.K.Y., Brett K.R. (2018). What Happens to Patients When We Do Not Repair Their Cuff Tears? Five-Year Rotator Cuff Quality-of-Life Index Outcomes Following Nonoperative Treatment of Patients with Full-Thickness Rotator Cuff Tears. J. Shoulder Elb. Surg..

[146] Paribelli G., Boschi S., Randelli P., Compagnoni R., Leonardi F., Cassarino A.M. (2015). Clinical Outcome of Latissimus Dorsi Tendon Transfer and Partial Cuff Repair in Irreparable Postero-Superior Rotator Cuff Tear. Musculoskelet. Surg..

[147] Mohtadi N.G., Hollinshead R.M., Sasyniuk T.M., Fletcher J.A., Chan D.S., Li F.X. (2008). A Randomized Clinical Trial Comparing Open to Arthroscopic Acromioplasty with Mini-Open Rotator Cuff Repair for Full-Thickness Rotator Cuff Tears: Disease-Specific Quality of Life Outcome at an Average 2-Year Follow-Up. Am. J. Sports Med..

[148] Papalia R., Osti L., Leonardi F. (2010). RC-QOL Score for Rotator Cuff Pathology: Adaptation to Italian. Knee Surg. Sport. Traumatol. Arthrosc..

[149] Wang W., Zhang C., Cui L., Xie Q., Jia Z., Zheng W. (2018). Reliability, Validity and Responsiveness of the Chinese Version of the Rotator Cuff Quality of Life Index (RC-QOL) in Patients with Rotator Cuff Disorders. PLoS ONE.

[150] Li H.M., Chau J.Y.M., Woo S.B., Lai J., Chan W.L. (2020). Chinese Version of the Rotator Cuff Quality of Life Questionnaire: Cross-Cultural Adaptation and Validation in Rotator Cuff-Impaired Patients in Hong Kong. J. Orthop. Trauma Rehabil..

[151] Gunes T., Erkorkmaz U., Kurnaz R., Bilgic E., Asci M. (2015). Rotator Cuff—Quality of Life Scale: Adaptation to Turkish. Knee Surg. Sport. Traumatol. Arthrosc..

[152] Huber W., Hofstaetter J.G., Hanslik-Schnabel B., Posch M., Wurnig C. (2005). Übersetzung Und Psychometrische Austestung Des Rotator Cuff Quality-of-Life Measure (RC-QOL) Für Den Gebrauch Im Deutschen Sprachraum. Z. Rheumatol..

[153] Rodríguez L.R., Izquierdo T.G., Martín D.P. (2020). Adaptation and Transcultural Translation of the Rotator Cuff Quality of Life Questionnaire into Spanish. J. Shoulder Elb. Surg..

[154] Sebastiá-Forcada E., Martínez-Rico S., Vizcaya-Moreno M.F., Lizaur-Utrilla A. (2019). Prospective Study on Effectiveness and Safety of Arthroscopic Bankart Using a Single Anterior Portal for Patients with Anterior Shoulder Instability. Rev. Esp. Cir. Ortop. Traumatol..

[155] Barnes C.J., Getelman M.H., Snyder S.J. (2009). Results of Arthroscopic Revision Anterior Shoulder Reconstruction. Am. J. Sports Med..

[156] Ikemoto R.Y., Murachovisky J., Nascimento L.G.P., Bueno R.S., Almeida L.H.O., Strose E., Helmer F.F. (2011). Results From Latarjet Surgery for Treating Traumatic Anterior Shoulder Instability Associated With Bone Erosion in the Glenoid Cavity, After Minimum Follow-Up of One Year. Rev. Bras. Ortop..

[157] García-Rodíguez R., Díez-Nicolás E., Vilá-y-Rico J., Martín-López C.M., Cano-Egea J.M. (2011). Resultados a Mediano Plazo de La Reparación Artroscópica En La Inestabilidad Recidivante Glenohumeral Anteroinferior. Acta Ortopédica Mex..

[158] Marcondes F.B., de Vasconcelos R.A., Marchetto A., de Andrade A.L.L., Filho A.Z., Etchebehere M. (2012). Translation and Cross-Cultural Adaptation of the Rowe Score for Portuguese. Acta Ortop. Bras..

[159] Razmjou H., Stratford P., Holtby R. (2012). A Shortened Version of the Western Ontario Rotator Cuff Disability Index: Development and Measurement Properties. Physiother. Canada.

[160] Dewan N., MacDermid J.C., MacIntyre N., Grewal R. (2016). Reproducibility: Reliability and Agreement of Short Version of Western Ontario Rotator Cuff Index (Short-WORC) in Patients with Rotator Cuff Disorders. J. Hand Ther..

[161] Dewan N., MacDermid J.C., MacIntyre N., Grewal R. (2018). Validity and Responsiveness of the Short Version of the Western Ontario Rotator Cuff Index (Short-WORC) in Patients with Rotator Cuff Repair. J. Orthop. Sport. Phys. Ther..

[162] Furtado R., MacDermid J.C., Bryant D.M., Faber K.J., Athwal G.S. (2020). Interpretation and Content Validity of the Items of the Numeric Rating Version Short-WORC to Evaluate Outcomes in Management of Rotator Cuff Pathology: A Cognitive Interview Approach. Health Qual. Life Outcomes.

[163] Negahban H., Mohtasebi E., Goharpey S. (2015). Reliability, Validity, and Responsiveness of the Persian Version of Shoulder Activity Scale in a Group of Patients with Shoulder Disorders. Disabil. Rehabil..

[164] Edwards P.K., Ebert J.R., Morrow M.M., Goodwin B.M., Ackland T., Wang A. (2020). Accelerometry Evaluation of Shoulder Movement and Its Association with Patient-Reported and Clinical Outcomes Following Reverse Total Shoulder Arthroplasty. J. Shoulder Elb. Surg..

[165] Van de Water A.T.M., Davidson M., Shields N., Evans M.C., Taylor N.F. (2015). The Shoulder Function Index (SFInX): A Clinician-Observed Outcome Measure for People with a Proximal Humeral Fracture. BMC Musculoskelet. Disord..

[166] Van de Water A.T.M., Davidson M., Shields N., Evans M.C., Taylor N.F. (2016). The Shoulder Function Index (SFInX): Evaluation of Its Measurement Properties in People Recovering from a Proximal Humeral Fracture. BMC Musculoskelet. Disord..

[167] Roach K.E., Budiman-Mak E., Songsiridej N., Lertratanakul Y. (1991). Development of a Shoulder Pain and Disability Index. Arthritis Rheum..

[168] Heald S.L., Riddle D.L., Lamb R.L. (1997). The Shoulder Pain and Disability Index: The Construct Validity and Responsiveness of a Region-Specific Disability Measure. Phys. Ther..

[169] Roddey T.S., Olson S.L., Cook K.F., Gartsman G.M., Hanten W. (2000). Comparison of the University of California—Los Angeles Shoulder Scale and the Simple Shoulder Test with the Shoulder Pain and Disability Index: Single-Administration Reliability and Validity. Phys. Ther..

[170] MacDermid J.C., Solomon P., Prkachin K. (2006). The Shoulder Pain and Disability Index Demonstrates Factor, Construct and Longitudinal Validity. BMC Musculoskelet. Disord..

[171] Bicer A., Ankarali H. (2010). Shoulder Pain and Disability Index: A Validation Study in Turkish Women. Singapore Med. J..

[172] Staples M.P., Forbes A., Green S., Buchbinder R. (2010). Shoulder-Specific Disability Measures Showed Acceptable Construct Validity and Responsiveness. J. Clin. Epidemiol..

[173] Hill C.L., Lester S., Taylor A.W., Shanahan M.E., Gill T.K. (2011). Factor Structure and Validity of the Shoulder Pain and Disability Index in a Population-Based Study of People with Shoulder Symptoms. BMC Musculoskelet. Disord..

[174] Riley S.P., Cote M.P., Swanson B., Tafuto V., Sizer P.S., Brismée J.M. (2015). The Shoulder Pain and Disability Index: Is It Sensitive and Responsive to Immediate Change?. Man. Ther..

[175] Jerosch-Herold C., Chester R., Shepstone L., Vincent J.I., MacDermid J.C. (2018). An Evaluation of the Structural Validity of the Shoulder Pain and Disability Index (SPADI) Using the Rasch Model. Qual. Life Res..

[176] Thoomes-De Graaf M., Scholten-Peeters W., Duijn E., Karel Y., de Vet H.C.W., Koes B., Verhagen A. (2017). The Responsiveness and Interpretability of the Shoulder Pain and Disability Index. J. Orthop. Sports Phys. Ther..

[177] Riley S.P., Tafuto V., Cote M., Brismée J.M., Wright A., Cook C. (2019). Reliability and Relationship of the Fear-Avoidance Beliefs Questionnaire with the Shoulder Pain and Disability Index and Numeric Pain Rating Scale in Patients with Shoulder Pain. Physiother. Theory Pract..

[178] Boake B.R., Childs T.K., Soules T.D., Zervos D.L., Vincent J.I., MacDermid J.C. (2020). Rasch Analysis of The Shoulder Pain and Disability Index (SPADI) in a Postrepair Rotator Cuff Sample. J. Hand Ther..

[179] Angst F., Goldhahn J., Pap G., Mannion A.F., Roach K.E., Siebertz D., Drerup S., Schwyzer H.K., Simmen B.R. (2007). Cross-Cultural Adaptation, Reliability and Validity of the German Shoulder Pain and Disability Index (SPADI). Rheumatology.

[180] Alsanawi H.A., Alghadir A., Anwer S., Roach K.E., Alawaji A. (2015). Cross-Cultural Adaptation and Psychometric Properties of an Arabic Version of the Shoulder Pain and Disability Index. Int. J. Rehabil. Res..

[181] Wang W., Jia Z.-Y., Liu J., Xie Q.-Y., Cui J., Zheng W., Xu W.-D. (2018). Cross-Cultural Adaptation and Validation of the Chinese Version of the Shoulder Pain and Disability Index in Patients with Symptomatic Shoulder Pain: A Prospective Case Series. Medicine.

[182] Yao M., Yang L., Cao Z.Y., Cheng S.D., Tian S.L., Sun Y.L., Wang J., Xu B.P., Hu X.C., Wang Y.J. (2017). Translation and Cross-Cultural Adaptation of the Shoulder Pain and Disability Index (SPADI) into Chinese. Clin. Rheumatol..

[183] Christensen K.B., Thorborg K., Holmich P., Clausen M.B. (2019). Rasch Validation of the Danish Version of the Shoulder Pain and Disability Index (SPADI) in Patients with Rotator Cuff-Related Disorders. Qual. Life Res..

[184] Thoomes-de Graaf M., Scholten-Peeters G.G., Duijn E., Karel Y., Koes B.W., Verhagen A.P. (2015). The Dutch Shoulder Pain and Disability Index (SPADI): A reliability and validation study. Qual Life Res..

[185] Vrouva S., Batistaki C., Koutsioumpa E., Kostopoulos D., Stamoulis E., Kostopanagiotou G. (2016). The Greek Version of Shoulder Pain and Disability Index (SPADI): Translation, Cultural Adaptation, and Validation in Patients with Rotator Cuff Tear. J. Orthop. Traumatol..

[186] Spanou A., Mamais I., Lamnisos D., Stasinopoulos D. (2020). Reliability and Validity of the Greek Shoulder Pain and Disability Index in Patients with Shoulder Pain. Disabil. Rehabil..

[187] Brindisino F., Indaco T., Giovannico G., Ristori D., Maistrello L., Turolla A. (2020). Shoulder Pain and Disability Index: Italian Cross-Cultural Validation in Patients with Non-Specific Shoulder Pain. Shoulder Elb..

[188] Choi Y., Park J.W., Noh S., Kim M.S., Park Y.H., Sung D.H. (2015). Reliability, Validity, and Responsiveness of the Korean Version of the Shoulder Disability Questionnaire and Shoulder Rating Questionnaire. Ann. Rehabil. Med..

[189] Sudarshan K.C., Sharma S., Ginn K., Almadi T., Subedi H., Reed D., Sudarshan K.C., Sharma S., Ginn K., Almadi T. (2019). Cross-Cultural Adaptation and Measurement Properties of the Nepali Version of the DASH (Disability of Arm, Shoulder and Hand) in Patients with Shoulder Pain. Health Qual. Life Outcomes.

[190] Jamnik H., Spevak M.K. (2008). Shoulder Pain and Disability Index: Validation of Slovene Version. Int. J. Rehabil. Res..

[191] Phongamwong C., Choosakde A. (2015). Reliability and Validity of the Thai Version of the Shoulder Pain and Disability Index (Thai SPADI). Health Qual. Life Outcomes.

[192] Gadam Y.K., Subramanian S., Patchava A., Kumar S.C., Neerukonda S.J., Kambarthi N. (2018). Reliability and Validity of the Indian (Telugu) Version of the Shoulder Pain and Disability Index. J. Clin. Diagnostic Res..

[193] Sekiguchi T., Hagiwara Y., Ando A., Kanazawa K., Suzuki K., Koide M., Yabe Y., Onoda S., Itoi E. (2020). Validation and Reliability of a Japanese Version of the Shoulder Pain and Disability Index: A Cross-Sectional Study. J. Orthop. Sci..

[194] Luque-Suarez A., Rondon-Ramos A., Fernandez-Sanchez M., Roach K.E., Morales-Asencio J.M. (2016). Spanish Version of SPADI (Shoulder Pain and Disability Index) in Musculoskeletal Shoulder Pain: A New 10-Items Version after Confirmatory Factor Analysis. Heal. Qual. Life Outcomes.

[195] Breckenridge J.D., McAuley J.H. (2011). Shoulder Pain and Disability Index (SPADI). J. Physiother..

[196] Winters J.C., Sobel J.S., Groenier K.H., Arendzen J.H., Meyboom-De Jon B. (1996). A Shoulder Pain Score: A Comprehensive Questionnaire for Assessing Pain in Patients with Shoulder Complaints. Scand. J. Rehabil. Med..

[197] Sicard J., Klouche S., Conso C., Billot N., Auregan J.-C., Poulain S., Lespagnol F., Solignac N., Bauer T., Ferrand M. (2019). Local Infiltration Analgesia versus Interscalene Nerve Block for Postoperative Pain Control after Shoulder Arthroplasty: A Prospective, Randomized, Comparative Noninferiority Study Involving 99 Patients. J. Shoulder Elb. Surg..

[198] Heller B., Tarricone R. (2004). Oxaprozin versus Diclofenac in NSAID-Refractory Periarthritis Pain of the Shoulder. Curr. Med. Res. Opin..

[199] Desroches A., Klouche S., Schlur C., Bauer T., Waitzenegger T., Hardy P. (2016). Suprascapular Nerve Block Versus Interscalene Block as Analgesia After Arthroscopic Rotator Cuff Repair: A Randomized Controlled Noninferiority Trial. Arthrosc. J. Arthrosc. Relat. Surg..

[200] Penning L.I.F., De Bie R.A., Walenkamp G.H.I.M. (2012). The Effectiveness of Injections of Hyaluronic Acid or Corticosteroid in Patients with Subacromial Impingement: A Three-Arm Randomised Controlled Trial. J. Bone Jt. Surg. Ser. B.

[201] Champion J.K., Williams M. (2006). Prospective Randomized Trial of Heated Humidified versus Cold Dry Carbon Dioxide Insufflation during Laparoscopic Gastric Bypass. Surg. Obes. Relat. Dis..

[202] Fan S., Liang F.Y., Chen W.L., Yang Z.H., Huang X.M., Wang Y.Y., Lin Z.Y., Zhang D.M., Zhou B., Chen W.X. (2014). Minimally Invasive Selective Neck Dissection: A Prospective Study of Endoscopically Assisted Dissection via a Small Submandibular Approach in CT1–2N0Oral Squamous Cell Carcinoma. Ann. Surg. Oncol..

[203] L’ insalata J.C., Warren R.F., Cohen S.B., Altchek D.W., Peterson M.G. (1997). Self-Administered Questionnaire for Assessment of Symptoms and Function of the Shoulder. J. Bone Jt. Surg..

[204] Vermeulen H.M., Boonman D.C.G., Schüller H.M., Obermann W.R., van Houwelingen H.C., Rozing P.M., Vliet Vlieland T.P.M. (2005). Translation, Adaptation and Validation of the Shoulder Rating Questionnaire (SRQ) into the Dutch Language. Clin. Rehabil..

[205] De Siqueira D.C., Baptista A.F., Souza I., Sád K.N. (2014). Tradução, Adaptação Cultural, Validade e Confiabilidade Do Questionário de Classificação Do Ombro Para Uso No Brasil. Rev. Bras. Reumatol..

[206] Lippitt S.B., Harryman D.T., Matsen F. (1993). A Practical Tool for Evaluation of Th Function: The Simple Shoulder Test. Acad. Orthop. Surg..

[207] Godfrey J., Hamman R., Lowenstein S., Briggs K., Kocher M. (2007). Reliability, Validity, and Responsiveness of the Simple Shoulder Test: Psychometric Properties by Age and Injury Type. J. Shoulder Elb. Surg..

[208] Roy J.S., MacDermid J.C., Faber K.J., Drosdowech D.S., Athwal G.S. (2010). The Simple Shoulder Test Is Responsive in Assessing Change Following Shoulder Arthroplasty. J. Orthop. Sports Phys. Ther..

[209] Hsu J.E., Russ S.M., Somerson J.S., Tang A., Warme W.J., Matsen F.A. (2017). Is the Simple Shoulder Test a Valid Outcome Instrument for Shoulder Arthroplasty?. J. Shoulder Elb. Surg..

[210] Greiwe R.M., Kohrs B.J., Callegari J., Harm R.G., Hill M.A., Boyle M.S. (2020). Open Reduction Internal Fixation vs. Reverse Shoulder Arthroplasty for the Treatment of Acute Displaced Proximal Humerus Fractures. Semin. Arthroplasty.

[211] Van Kampen D.A., van Beers L.W., Scholtes V.A., Terwee C.B., Willems W.J. (2012). Validation of the Dutch Version of the Simple Shoulder Test. J. Shoulder Elb. Surg..

[212] Naghdi S., Nakhostin Ansari N., Rustaie N., Akbari M., Ebadi S., Senobari M., Hasson S. (2015). Simple Shoulder Test and Oxford Shoulder Score: Persian Translation and Cross-Cultural Validation. Arch. Orthop. Trauma Surg..

[213] Osni J., Neto B., Gesser R.L., Steglich V., Bonilauri A.P., Vissoci N., Pietrobon R. (2013). Validation of the Simple Shoulder Test in a Portuguese- Brazilian Population. Is the Latent Variable Structure and Validation of the Simple Shoulder Test Stable across Cultures?. PLoS ONE.

[214] Ryliskis S., Piesina E., Kocius M., Marx R.G. (2008). Cross-Cultural Adaptation and Psychometric Properties of the Lithuanian Version of the Simple Shoulder Test. Acta Med. Litu..

[215] Membrilla-Mesa M.D., Tejero-Fernández V., Cuesta-Vargas A.I., Arroyo-Morales M. (2015). Validation and Reliability of a Spanish Version of Simple Shoulder Test (SST-Sp). Qual. Life Res..

[216] Williams G.N., Gangel T.J., Arciero R.A., Uhorchak J.M., Taylor D.C. (1999). Comparison of the Single Assessment Numeric Evaluation Method and Two Shoulder Rating Scales. Outcomes Measures after Shoulder Surgery. Am. J. Sports Med..

[217] Gowd A.K., Charles M.D., Liu J.N., Lalehzarian S.P., Cabarcas B.C., Manderle B.J., Nicholson G.P., Romeo A.A., Verma N.N. (2019). Single Assessment Numeric Evaluation (SANE) Is a Reliable Metric to Measure Clinically Significant Improvements Following Shoulder Arthroplasty. J. Shoulder Elb. Surg..

[218] Thigpen C.A., Shanley E., Momaya A.M., Kissenberth M.J., Tolan S.J., Tokish J.M., Hawkins R.J. (2018). Validity and Responsiveness of the Single Alpha-Numeric Evaluation for Shoulder Patients. Am. J. Sports Med..

[219] Cohn M.R., Kunze K.N., Polce E.M., Nemsick M., Garrigues G.E., Forsythe B., Nicholson G.P., Cole B.J., Verma N.N. (2020). Establishing Clinically Significant Outcome Thresholds for the Single Assessment Numeric Evaluation 2 Years Following Total Shoulder Arthroplasty. J. Shoulder Elb. Surg..

[220] Baumgarten K.M., Osborn R., Schweinle W.E., Zens M.J. (2018). The Influence of Anatomic Total Shoulder Arthroplasty Using a Subscapularis Tenotomy on Shoulder Strength. J. Shoulder Elb. Surg..

[221] Khazzam M.S., Mulligan E., Shirley Z., Brunette M. (2015). Sleep Quality in Patients with Rotator Cuff Disease. Orthop. J. Sport. Med..

[222] Kohan E.M., Wong J., Stroh M., Syed U.A.M., Namdari S., Lazarus M. (2020). Outcome of Biceps Suspensionplasty for Recurrent Multidirectional Shoulder Instability. J. Orthop..

[223] Kohn D., Geyer M., Wülker N. The Subjective Shoulder Rating Scale (SSRS)—An Examiner-Independent Scoring System. Proceedings of the International Congress on Surgery of the Shoulder.

[224] Kohn D., Geyer M. (1997). The Subjective Shoulder Rating System. Arch. Orthop. Trauma Surg..

[225] Amstutz H.C., Sew Hoy A.L., Clarke I.C. (1981). UCLA Anatomic Total Shoulder Arthroplasty. Clin. Orthop. Relat. Res..

[226] Akhtar M., Awaiz Nadeem R.D., Hassan Shah Gillani S.F.U., Cheema O.I., Nadeem M.R. (2019). Comparison of Intra Articular NSAID (Ketorolac) Injection versus Hyaluronic Acid Injection for the Mean Decrease of Pain Score (According to UCLA Shoulder Rating Scale) in the Management of Adhesive Capsulitis. Pak. J. Pharm. Sci..

[227] Rompe J.D., Zoellner J., Nafe B. (2001). Shock Wave Therapy versus Conventional Surgery in the Treatment of Calcifying Tendonitis of the Shoulder. Clinical Orthopedics and Related Research. Clin. Orthop. Relat. Res..

[228] Bosch U., Skutek M., Fremerey R.W., Tscherne H. (1998). Outcome after Primary and Secondary Hemiarthroplasty in Elderly Patients with Fractures of the Proximal Humerus. J. Shoulder Elb. Surg..

[229] Lim W.J., Rahmatullah H., Abd B., Lim L., Dhanaraj I.D., Mohamed S., Mosaid S., Tan H.A. (2015). Outcomes Are Favorable in Asian Patients Undergoing Deltoid-on Open Rotator Cuff Repair without Acromioplasty. J. Orthop..

[230] Croft P., Pope D., Zonca M., Neill T.O., Silman A., O’Neill T., Silman A. (1994). Measurement of Shoulder Related Disability: Results of a Validation Study. Ann. Rheum. Dis..

[231] Brindisino F., Pellicciari L., Lorusso M., Pennella D., Padua R., Di Bari M. (2020). Cross-Cultural Adaptation, Reliability, and Validity of the Italian Version of the Shoulder Disability Questionnaire. Musculoskelet. Sci. Pract..

[232] Kirkley A., Griffin S., McLintock H., Ng L. (1998). The Development and Evaluation of a Disease-Specific Quality of Life Measurement Tool for Shoulder Instability: The Western Ontario Shoulder Instability Index (WOSI). Am. J. Sports Med..

[233] Provencher M.T., Frank R.M., Golijanin P., Gross D., Cole B.J., Verma N.N., Romeo A.A. (2017). Distal Tibia Allograft Glenoid Reconstruction in Recurrent Anterior Shoulder Instability: Clinical and Radiographic Outcomes. Arthrosc. J. Arthrosc. Relat. Surg..

[234] Hines A., Cook J.B., Shaha J.S., Krul K., Shaha S.H., Johnson J., Bottoni C.R., Rowles D.J., Tokish J.M. (2018). Glenoid Bone Loss in Posterior Shoulder Instability: Prevalence and Outcomes in Arthroscopic Treatment. Am. J. Sports Med..

[235] Skare Ø., Liavaag S., Reikerås O., Mowinckel P., Brox J.I. (2013). Evaluation of Oxford Instability Shoulder Score, Western Ontario Shoulder Instability Index and Euroqol in Patients with Slap (Superior Labral Anterior Posterior) Lesions or Recurrent Anterior Dislocations of the Shoulder. BMC Res. Notes.

[236] Gaudelli C., Balg F., Godbout V., Pelet S., Djahangiri A., Griffin S., Rouleau D.M. (2014). Validity, Reliability and Responsiveness of the French Language Translation of the Western Ontario Shoulder Instability Index (WOSI). Orthop. Traumatol. Surg. Res..

[237] Perrin C., Khiami F., Beguin L., Calmels P., Gresta G., Edouard P. (2017). Translation and Validation of the French Version of the Western Ontario Shoulder Instability Index (WOSI): WOSI-Fr. Orthop. Traumatol. Res..

[238] Eshoj H., Bak K., Blond L., Juul-Kristensen B. (2017). Translation, Adaptation and Measurement Properties of an Electronic Version of the Danish Western Ontario Shoulder Instability Index (WOSI). BMJ Open.

[239] Wiertsema S.H., De Witte P.B., Rietberg M.B., Hekman K.M., Schothorst M., Steultjens M.P., Dekker J. (2014). Measurement Properties of the Dutch Version of the Western Ontario Shoulder Instability Index (WOSI). J. Orthop. Sci..

[240] Van der Linde J.A., Willems W.J., van Kampen D.A., van Beers L.W., van Deurzen D.F., Terwee C.B. (2014). Measurement Properties of the Western Ontario Shoulder Instability Index in Dutch Patients with Shoulder Instability. BMC Musculoskelet. Disord..

[241] Hofstaetter J.G., Hanslik-Schnabel B., Hofstaetter S.G., Wurnig C., Huber W. (2010). Cross-Cultural Adaptation and Validation of the German Version of the Western Ontario Shoulder Instability Index. Arch. Orthop. Trauma Surg..

[242] Gottlieb U., Springer S. (2019). Translation and Validation of a Hebrew Version of the Western Ontario Shoulder Instability Index. J. Orthop. Surg. Res..

[243] Cacchio A., Paoloni M., Griffin S.H., Rosa F., Properzi G., Padua L., Padua R., Carnelli F., Calvisi V., Santilli V. (2012). Cross-Cultural Adaptation and Measurement Properties of an Italian Version of the Western Ontario Shoulder Instability Index (WOSI). J. Orthop. Sports Phys. Ther..

[244] Hatta T., Shinozaki N., Omi R., Sano H., Yamamoto N., Ando A., Sugaya H., Aizawa T., Kuriyama S., Itoi E. (2011). Reliability and Validity of the Western Ontario Shoulder Instability Index (WOSI) in the Japanese Population. J. Orthop. Sci..

[245] Salomonsson B., Ahlström S., Dalén N., Lillkrona U. (2009). The Western Ontario Shoulder Instability Index (WOSI): Validity, Reliability, and Responsiveness Retested with a Swedish Translation. Acta Orthop..

[246] Basar S., Gunaydin G., Kanik Z.H., Sozlu U., Alkan Z.B., Pala O.O., Citaker S., Kanatli U. (2017). Western Ontario Shoulder Instability Index: Cross-Cultural Adaptation and Validation of the Turkish Version. Rheumatol. Int..

[247] Khaja D.A., Bouhamra D.A., Hanna D.S., Maqdis D.A. (2020). Cross-Cultural Adaptation and Psychometric Properties of an Arabic Version of the Western Ontario Shoulder Instability Index (WOSI). Int. J. Inn. Res. Med. Sci..

[248] Ismail M.M., El Shorbagy K.M., Mohamed A.R., Griffin S.H. (2020). Cross-Cultural Adaptation and Validation of the Arabic Version of the Western Ontario Shoulder Instability Index (WOSI-Arabic). Orthop. Traumatol. Surg. Res..

[249] Yuguero M., Huguet J., Griffin S.J., Sirvent Ribalda E., Marcano Fernández F.A., Balaguer Castro M., Torner Pifarré P. (2016). Adaptación Transcultural, Validación y Valoración de Las Propiedades Psicométricas, de La Versión Española Del Cuestionario Western Ontario Shoulder Instability Index. Rev. Esp. Cir. Ortop. Traumatol..

[250] Lo I.K.Y., Griffin S., Kirkley A. (2001). The Development of a Disease-Specific Quality of Life Measurement Tool for Osteoarthritis of the Shoulder: The Western Ontario Osteoarthritis of the Shoulder (WOOS) Index. Osteoarthr. Cartil..

[251] Saad M.A., Kassam H.F., Suriani R.J., Pan S.D., Blaine T.A., Kovacevic D. (2018). Performance of PROMIS Global-10 Compared with Legacy Instruments in Patients with Shoulder Arthritis. J. Shoulder Elb. Surg..

[252] Baumgarten K.M., Chang P.S., Dannenbring T.M., Foley E.K. (2018). Does Total Shoulder Arthroplasty Improve Patients’ Activity Levels?. J. Shoulder Elb. Surg..

[253] Mannberg Backman S., Strat S., Ahlstrom S., Brodin N. (2016). Validity and Sensitivity to Change of the Patient Specific Functional Scale Used during Rehabilitation Following Proximal Humeral Fracture. Disabil. Rehabil..

[254] Rasmussen J., Jakobsen J., Olsen B.S., Brorson S. (2013). Translation and Validation of the Western Ontario Osteoarthritis of the Shoulder (WOOS) index – the Danish version. Patient Relat. Outcome Meas..

[255] Corona K., Cerciello S., Morris B.J., Visonà E., Merolla G., Porcellini G. (2016). Cross-Cultural Adaptation and Validation of the Italian Version of the Western Ontario Osteoarthritis of the Shoulder Index (WOOS). J. Orthop. Traumatol..

[256] Klintberg I.H., Lind K., Marlow T., Svantesson U. (2012). Western Ontario Osteoarthritis Shoulder (WOOS) Index: A Cross-Cultural Adaptation into Swedish, Including Evaluation of Reliability, Validity, and Responsiveness in Patients with Subacromial Pain. J. Shoulder Elb. Surg..

[257] Jia Z., Zhang C., Cui J., Xue C., Xu W. (2018). Translation and Validation of the Simplified Chinese Version of Western Ontario Osteoarthritis of the Shoulder Index (WOOS). Medicine.

[258] Kirkley A., Alvarez C., Griffin S. (2003). The Development and Evaluation of a Disease-Specific Quality-of-Life Questionnaire for Disorders of the Rotator Cuff: The Western Ontario Rotator Cuff Index. Clin. J. Sport Med..

[259] Gadsboell J., Tibaek S. (2017). Validity of a Shoulder-Specific Quality of Life Questionnaire, the Western Ontario Rotator Cuff Index, for Patients with Scapula Alata. JSES Open Access.

[260] Nicholson A.D., Kassam H.F., Pan S.D., Berman J.E., Blaine T.A., Kovacevic D. (2019). Performance of PROMIS Global-10 Compared with Legacy Instruments for Rotator Cuff Disease. Am. J. Sports Med..

[261] Başkurt Z., Başkurt F., Gelecek N., Özkan M.H. (2011). The Effectiveness of Scapular Stabilization Exercise in the Patients with Subacromial Impingement Syndrome. J. Back Musculoskelet. Rehabil..

[262] Lopes A.D., Ciconelli R.M., Carrera E.F., Griffin S., Faloppa F., Dos Reis F.B. (2008). Validity and Reliability of the Western Ontario Rotator Cuff Index (WORC) for Use in Brazil. Clin. J. Sport Med..

[263] Wang W., Xie Q., Jia Z., Cui L., Liu D., Wang C., Zheng W. (2017). Cross-Cultural Translation of the Western Ontario Cuff Index in Chinese and Its Validation in Patients with Rotator Cuff Disorders. BMC Musculoskelet. Disord..

[264] Wessel R.N., Wolterbeek N., Fermont A.J.M., Van Mameren H., Sonneveld H., Griffin S., De Bie R.A. (2013). The Conceptually Equivalent Dutch Version of the Western Ontario Rotator Cuff Index (WORC)©. BMC Musculoskelet. Disord..

[265] Wiertsema S.H., Rietberg M.B., Hekman K.M., Schothorst M., Steultjens M.P., Dekker J. (2013). Reproducibility of the Dutch Version of the Western Ontario Rotator Cuff Index. J. Shoulder Elb. Surg..

[266] Kawabata M., Miyata T., Nakai D., Sato M., Tatsuki H., Kashiwazaki Y., Saito H. (2013). Reproducibility and Validity of the Japanese Version of the Western Ontario Rotator Cuff Index. J. Orthop. Sci..

[267] Mousavi S.J., Hadian M.R., Abedi M., Montazeri A. (2009). Translation and Validation Study of the Persian Version of the Western Ontario Rotator Cuff Index. Clin. Rheumatol..

[268] El O., Bircan C., Gulbahar S., Demiral Y., Sahin E., Baydar M., Kizil R., Griffin S., Akalin E. (2006). The Reliability and Validity of the Turkish Version of the Western Ontario Rotator Cuff Index. Rheumatol. Int..

[269] Brix L.D., Bjørnholdt K.T., Nikolajsen L., Kallestrup K., Thillemann T.M. (2020). The Danish Version of the Western Ontario Rotator Cuff Index. Dan. Med. J..

[270] St-Pierre C., Dionne C.E., Desmeules F., Roy J.S. (2015). Reliability, Validity, and Responsiveness of a Canadian French Adaptation of the Western Ontario Rotator Cuff (WORC) Index. J. Hand Ther..

[271] Bejer A., Probachta M., Kulczyk M., Griffin S., Domka-Jopek E., Płocki J., Probachta M., Domka-Jopek E., Płocki J. (2018). Validation of the Polish Version of the Western Ontario Rotator Cuff Index in Patients Following Arthroscopic Rotator Cuff Repair. BMC Musculoskelet. Disord..

[272] Zhaeentan S., Legeby M., Ahlström S., Stark A., Salomonsson B. (2016). A Validation of the Swedish Version of the WORC Index in the Assessment of Patients Treated by Surgery for Subacromial Disease Including Rotator Cuff Syndrome. BMC Musculoskelet. Disord..

[273] Prinsen C.A.C., Mokkink L.B., Bouter L.M., Alonso J., Patrick D.L., De Vet H.C.W., Terwee C.B. (2018). COSMIN guideline for systematic reviews of patient-reported outcome measures. Qual. Life Res..

[274] Terwee C.B., Mokkink L.B., Knol D.L., Ostelo R.W.J.G., Bouter L.M., De Vet H.C.W. (2012). Rating the Methodological Quality in Systematic Reviews of Studies on Measurement Properties: A Scoring System for the COSMIN Checklist. Qual. Life Res..

[275] Noordzij M., Dekker F.W., Zoccali C., Jager K.J. (2011). Sample Size Calculations. Nephron Clin. Pract..

[276] Tashjian R.Z. (2012). Epidemiology, Natural History, and Indications for Treatment of Rotator Cuff Tears. Clin. Sports Med..

[277] Erickson B.J., Shishani Y., Bishop M.E., Romeo A.A., Gobezie R. (2019). Adhesive Capsulitis: Demographics and Predictive Factors for Success Following Steroid Injections and Surgical Intervention. Arthrosc. Sport. Med. Rehabil..

[278] Dominguez-Romero J.G., Jimenez-Rejano J.J., Ridao-Fernández C., Chamorro-Moriana G. (2021). Exercise-Based Muscle Development Programs and Their Effectiveness in the Functional Recovery of Rotator Cuff Tendinopathy: A Systematic Review and Meta-Analysis. Diagnostics.

[279] Nelson E.C., Eftimovska E., Lind C., Hager A., Wasson J.H., Lindblad S. (2015). Patient Reported Outcome Measures in Practice. BMJ.

[280] Katz S. (1983). Assessing Self-Maintenance: Activities of Daily Living, Mobility, and Instrumental Activities of Daily Living. J. Am. Geriatr. Soc..

[281] Luime J.J., Koes B.W., Hendriksen I.J.M., Burdorf A., Verhagen A.P., Miedema H.S., Verhaar J.A.N. (2004). Prevalence and Incidence of Shoulder Pain in the General Population; a Systematic Review. Scand. J. Rheumatol..

[282] Challoumas D., Stavrou A., Dimitrakakis G. (2017). The Volleyball Athlete’s Shoulder: Biomechanical Adaptations and Injury Associations. Sport. Biomech..

[283] Struyf F., Tate A., Kuppens K., Feijen S., Michener L.A. (2017). Musculoskeletal Dysfunctions Associated with Swimmers’ Shoulder. Br. J. Sports Med..

[284] Zouzias I.C., Hendra J., Stodelle J., Limpisvasti O. (2018). Golf Injuries: Epidemiology, Pathophysiology, and Treatment. J. Am. Acad. Orthop. Surg..

[285] Hendriks S.M., Spijker J., Licht C.M.M., Hardeveld F., De Graaf R., Batelaan N.M., Penninx B.W.J.H., Beekman A.T.F. (2015). Long-Term Work Disability and Absenteeism in Anxiety and Depressive Disorders. J. Affect. Disord..

[286] Salles J.I., Velasques B., Cossich V., Nicoliche E., Ribeiro P., Amaral M.V., Motta G. (2015). Strength Training and Shoulder Proprioception. J. Athl. Train..

[287] Veeger H.E.J., van der Helm F.C.T. (2007). Shoulder Function: The Perfect Compromise between Mobility and Stability. J. Biomech..

[288] Martinez-Calderon J., Meeus M., Struyf F., Miguel Morales-Asencio J., Gijon-Nogueron G., Luque-Suarez A. (2018). The Role of Psychological Factors in the Perpetuation of Pain Intensity and Disability in People with Chronic Shoulder Pain: A Systematic Review. BMJ Open.

